# Genome-wide analysis identifies gain and loss/change of function within the small multigenic insecticidal Albumin 1 family of *Medicago truncatula*

**DOI:** 10.1186/s12870-016-0745-0

**Published:** 2016-03-10

**Authors:** L. Karaki, P. Da Silva, F. Rizk, C. Chouabe, N. Chantret, V. Eyraud, F. Gressent, C. Sivignon, I. Rahioui, D. Kahn, C. Brochier-Armanet, Y. Rahbé, C. Royer

**Affiliations:** INRA, UMR0203 BF2I, Biologie Fonctionnelle Insectes et Interactions, F-69621 Villeurbanne, France; Insa-Lyon, UMR0203 BF2I, F-69621 Villeurbanne, France; ER030-EDST; Department of Life and Earth Sciences, Faculty of Sciences II, Lebanese University, Beirut, Lebanon; Université de Lyon, F-69000 Lyon, France; INRA, UMR1334 AGAP, 2 Place Pierre Viala, 34060 Montpellier, France; Supagro Montpellier, 2 Place Pierre Viala, 34060 Montpellier, France; UCBL, CarMeN Laboratory, INSERM UMR-1060, Cardioprotection Team, Faculté de Médecine, Univ Lyon-1, Université Claude Bernard Lyon1, 8 Avenue Rockefeller, 69373 Lyon Cedex 08, France; Université Claude Bernard Lyon 1; CNRS; INRA; UMR5558, Laboratoire de Biométrie et Biologie Evolutive, Université de Lyon, 43 boulevard du 11 novembre 1918, F-69622 Villeurbanne, France

**Keywords:** Legumes, Insecticidal protein, Insect-plant interaction, Cystine-knot peptides, Multigenic protein family evolution

## Abstract

**Background:**

Albumin 1b peptides (A1b) are small disulfide-knotted insecticidal peptides produced by Fabaceae (also called Leguminosae). To date, their diversity among this plant family has been essentially investigated through biochemical and PCR-based approaches. The availability of high-quality genomic resources for several fabaceae species, among which the model species *Medicago truncatula* (*Mtr*), allowed for a genomic analysis of this protein family aimed at *i)* deciphering the evolutionary history of A1b proteins and their links with A1b-nodulins that are short non-insecticidal disulfide-bonded peptides involved in root nodule signaling and *ii)* exploring the functional diversity of A1b for novel bioactive molecules.

**Results:**

Investigating the *Mtr* genome revealed a remarkable expansion, mainly through tandem duplications, of albumin1 (A1) genes, retaining nearly all of the same canonical structure at both gene and protein levels. Phylogenetic analysis revealed that the ancestral molecule was most probably insecticidal giving rise to, among others, A1b-nodulins. Expression meta-analysis revealed that many A1b coding genes are silent and a wide tissue distribution of the A1 transcripts/peptides within plant organs. Evolutionary rate analyses highlighted branches and sites with positive selection signatures, including two sites shown to be critical for insecticidal activity. Seven peptides were chemically synthesized and folded in vitro, then assayed for their biological activity. Among these, AG41 (*aka MtrA1013* isoform, encoded by the orphan TA24778 contig.), showed an unexpectedly high insecticidal activity. The study highlights the unique burst of diversity of A1 peptides within the *Medicago* genus compared to the other taxa for which full-genomes are available: no A1 member in *Lotus*, or in red clover to date, while only a few are present in chick pea, soybean or pigeon pea genomes.

**Conclusion:**

The expansion of the A1 family in the *Medicago* genus is reminiscent of the situation described for another disulfide-rich peptide family, the “Nodule-specific Cysteine-Rich” (NCR), discovered within the same species. The oldest insecticidal A1b toxin was described from the Sophorae, dating the birth of this seed-defense function to more than 58 million years, and making this model of plant/insect toxin/receptor (A1b/insect v-ATPase) one of the oldest known.

**Electronic supplementary material:**

The online version of this article (doi:10.1186/s12870-016-0745-0) contains supplementary material, which is available to authorized users.

## Background

Legumes (Fabaceae) are important economic crops that provide humans with food, livestock with feed and industry with raw materials [1]. Grain legume species, including pea (*Pisum sativum*), common bean (*Phaseolus vulgaris*), and lentil (*Lens culinaris*), account for over 33 % of human dietary protein. Other legumes, including clovers (*Trifolium* spp.) and medics (*Medicago* spp.), are widely used as animal fodder [2]. Many legumes have been used in folk medicine, indicative of their bioactive chemical diversity [3, 4]. They play a critical role in natural agricultural and forest ecosystems because of their position in the nitrogen cycle [5]. Due to this nodal ecological position, pests, being nitrogen-limited feeders, are a major constraint to legume production. They have consequently been involved in an evolutionary arms-race with legumes that defend themselves and their seeds through a wide array of chemical defenses and, remarkably, N-containing alkaloids, non-protein amino-acids and anti-nutritive peptides [6]. The isolation of legume peptides found to be acutely toxic for insect pests in stored vegetables and crops, and non-toxic to other taxa [7], has enlarged this defense arsenal, and, as a result, our possibilities for cereal grain protection [8, 9].

**Fig. 1:**
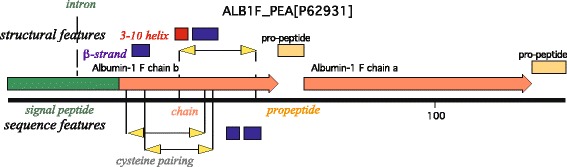
Peptide sequence features of the PA1 protein. All original Uniprot features of preproprotein PA1 (P62931, ALB1F_PEA) are displayed: Signal peptide shown in green (canonically interrupted by a short intron); mature PA1b toxin and PA1a proprotein are displayed as red arrows; processed propeptides are in *yellow boxes*; cysteine-pairing is represented by the *yellow arrows*. The β-strands are *boxed in blue* and the *3-* ^*10*^-helix in *red*. PA1b pertains to the Albumin I (IPR012512) Interpro family, which shows no relationship to other Interpro families

In plants as in animals, albumins (A) were defined by early biochemists as water soluble, moderately salt-soluble, and heat-denatured globular proteins. Plant albumins 1 (A1) are a technology-defined salt-soluble fraction from legume seed proteins, subsequently shown to be restricted to leguminous species in which they constitute the main supply of sulfur amino acids [10]. In pea seeds, the A1 gene, consisting of two exons and one intron, is transcribed as a single mRNA encoding the secreted polypeptide Pea Albumin 1 (PA1). The complex maturation of the latter finally leads to the release of two peptides, namely PA1b (4 kD) and PA1a (6 kD) (Fig. 1). To date, no function has been assigned to PA1a. The insect toxicity of PA1b was discovered in 1998 for weevils [8] and subsequently extended to numerous other insects [11]. In contrast to most animal venom toxins, it is active by ingestion, interacting with an intestinal binding site [12], recently identified as a V-ATPase proton pump [13]. PA1b consists of 37 amino acids with six cysteines involved in three disulfide bonds, ensuring a compact and stable structure to the toxin [8, 14], and belongs to the knottin structural group [15], The diversity of PA1b peptides within the same species was initially suggested by the work of Higgins et al. [16], which identified four functional genes that were present in the pea genome and expressed in pea seeds. Currently seven isoforms of PA1b have been isolated and biochemically characterized in the garden pea [8, 11–14], indicating that these peptides belong to a multigenic family whose members have diverged slightly [17]. More recently, a broad screen of more than 80 species scattered amongst the three major legume subfamilies identified ≈ 20 PA1-like genes from numerous Papilionoideae but none from Caesalpinioideae or Mimosoideae [18]. Thus, to date, the PA1b family seems to be strictly restricted to seeds of some legume sublineages and is the only one among more than 20 identified cysteine-rich families to have such a narrow distribution [19]. This suggests that the family may be an important line of high-N seed defense against insects. Recently an interesting case of horizontal transfer to a parasitic broomrape has been documented but does not alter the overall picture [20].

Although not the first plants to be subjected to genome sequencing, legumes are now included in genomic research specifically with soybean (*Glycine max*, Phaseoleae) and barrel medic (*Medicago truncatula*, Trifolieae) genome projects. The complete analysis of the *Medicago* genome for its rhizobial symbiotic features highlights this achievement [21]. The recent release of a very significantly improved genome assembly prompted us to conduct a genomic exploration of PA1b homologues within legumes, with the major aims of deciphering the evolutionary history of Albumins 1 and discovering new A1b variants with particular bioactivities. The study was mainly devoted to the six legume species whose full genome sequencing/assembly had been completed and publicly available [6 as of end 2014: *Medicago truncatula (Mtr*, Trifolieae*)* [21], *Glycine max (Gma*, Phaseoleae*)* [22], *Lotus japonicus (Lja*, Loteae*)* [23], *Cajanus cajan (Cca*, Phaseoleae*)* [24] *Cicer arietinum (Car*, Cicereae*)* [25] and *Phaseolus vulgaris (Pvu*, Phaseoleae*)* [26], plus that of *Trifolium pratense (Tpr*, Trifolieae*)* [27], still incompletely available. A specific focus was drawn on the model legume *M. truncatula* [21, 28]. In this species, despite the fact that no PA1b peptide was biochemically detected in the seeds, we had previously identified the presence of high insecticidal toxicity and of homologous genes in its genome [18].

## Results

### Specific expansion of the A1b family

**Fig. 2:**
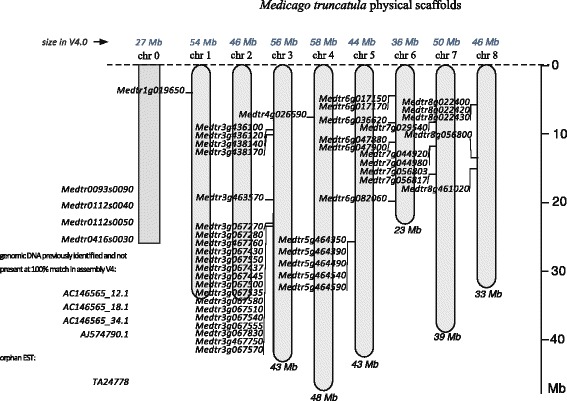
Organization of pa1 genes on Medicago truncatula genome. Positions of genes are indicated on chromosomes (scale in Mbp). The *Medicago truncatula* physical scaffold map is that of the genome assembly version 3.5 (including chromosome size and assembly quality map). Genes and their relative positions (%) on the chromosomes are those of assembly 4.1; a fictitious chromosome, called “0”, harbors the unplaced genes. AC146565_12.1, AC146565_18.1, AC146565_34.1 and AJ574790.1 are from genome version 3.5 and are not present at 100 % match in assembly v4. The orphan EST “TA24778_3880” is also reported

The survey of the *Medicago truncatula* genome (version 4.0v1 assembly) led to the identification of 52 A1 gene homologues. 44 genes were located on a *M. truncatula* chromosome (1 to 8), hence labeled Medtr*ng*, while eight genes were unassembled (four from the new V4.0 version: Medtr0093s0090, Medtr0112s0040, Medtr0112s0050 and Medtr 0416 s0030, and three were from the older V3.5 version: AC146565_12.1; AC146565_18.1; AC146565_34.1, plus the single, AJ574790.1 gene [18]; these were transiently located on a fictitious chromosome zero (Fig. 2, Additional file 1: Table S2). The detection of matching expressed sequence tags originating from *Mtr* databases (JCVI and Harvard Dana Farber repositories) showed that 22 of these genes are expressed (see § expression analysis). Finally, one EST sequence homologous to PA1 (TA24778@TIGR Plant Transcript Assemblies) could not be associated to any PA1 gene and thus remains orphan, bringing the total A1 gene family to 53 members. HMM profiles were constructed for both A1a and A1b families (ProDom families PDA1L0K4 and PD015795, respectively). A sensitive search of protein databases with these HMMs did not reveal significant relationships of these families outside the *Fabaceae.* Even the closely related structural family of cyclotides, bearing a similar cysteine topology (including CXC motif, see PFAM:PF03784), cannot be phylogenetically related to the albumins I.

The genomic organization and the structure of the *Mtr* A1 genes have been studied next. On the physical map, the 44 A1-encoding genes of *M. truncatula* are distributed on seven of the eight chromosomes, with an uneven distribution (from 1 to 21 genes per chromosome; Fig. 2). The length of all these genes varied between 470 and 1636 bp. Almost all genes (50/53) displayed a canonical organization with two exons and one intron. The latter was systematically positioned in the sequence coding for the signal peptide and its length varied between 87 and 1199 bp (Additional file 1: Table S2). Out of the 53 members, six forms seemed not to be secreted (no predicted signal peptide, Additional file 1: Table S2). Medtr8g056800.1 was the only gene harboring the C-terminal A1a subunit alone, and consequently also had no signal peptide. No trace of expression of this gene was found/published, questioning its functionality.

### Structural features of the *Medicago truncatula* peptide sequences

The characteristics of the 53 A1 candidates (including A1b peptide lengths, molecular masses and theoretical isoelectric points) are presented in additional files. All but 6 *M. truncatula* predicted peptides present a signal peptide 22–29 amino acids long, potentially leading the mature protein through the secretory/protein body pathway. The multiple alignment of PA1 proteins showed an overall higher conservation of A1a subunit compared to A1b (phylogeny section and Additional file 2: Table S5).

The location of the cysteine residues involved in the structural scaffolding of PA1b is globally conserved (Additional file 3: Table S3). More precisely four different cysteine organizations were observed. Typical A1b knottin (http://knottin.cbs.cnrs.fr, [29]) are characterized by six cysteine residues in a strongly conserved topology with an antepenultimate C_4_XC_5_ motif. 42 *Mtr* A1bs displayed this feature, whereas different patterns were observed for 6 A1b homologues (Additional file 3: Table S3). Cys6 was missing in the Medtr3g436120 encoded peptide, A1b from Medtr6g082060 and Medtr3g067830 harbored seven cysteines, and Medtr3g067430 and Medtr3g067445 held two additional cysteine residues after the Cys6.

### Phylogenetic analysis of *Medicago truncatula* A1bs

**Fig. 3:**
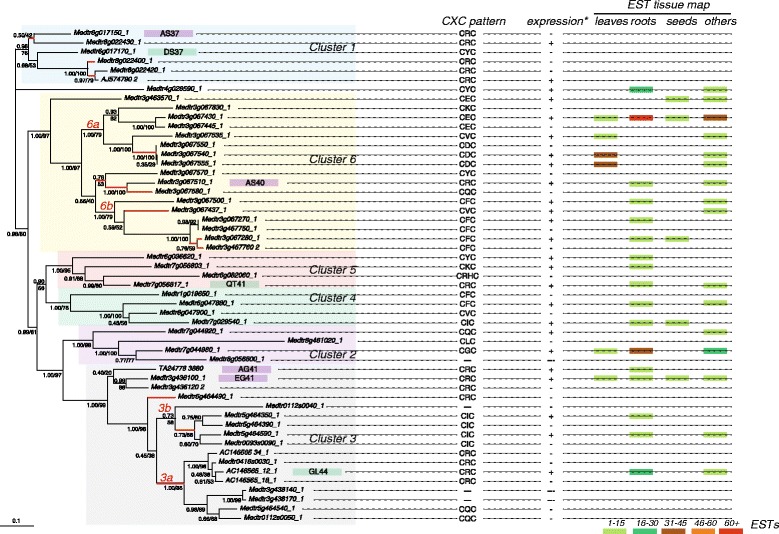
Phylogeny, CXC pattern and tissue EST expression of the *Medicago truncatula* PA1 paralogues. Unrooted Bayesian tree of *Medicago truncatula* PA1 family (53 sequences, 366 nucleic acid positions). The tree is presented according to rooted phylogeny shown on Fig. 4. Numbers at branch correspond respectively to posterior probabilities calculated with MrBayes, and to bootstrap values estimated by PhyML. The *scale bar* represents the average number of substitutions per site. Six strongly supported clusters were boxed and highlighted with colored backgrounds. The seven chemically and functionally synthetized sequences (AG41, EG41, GL44, AS40, AS37, DS37 and QT41) are indicated on the tree. Among them, AG41, EG41, AS40, and AS37 (*boxed in purple*), and showed toxicity against insect cells, whereas GL44, DS37 and QT41 (*boxed in light green*) did not show toxicity to insect cells. In the tissue expression part, the color-scale represent the expression value scales between 0 and >75 EST per cluster. Two internal sub-clusters were defined for running site model in cluster 3 and 6, and are named respectively 3a, 3b, and 6a, 6b (Table 2). Red branches are those that were tested for positive selection (Table 2)

**Fig. 4:**
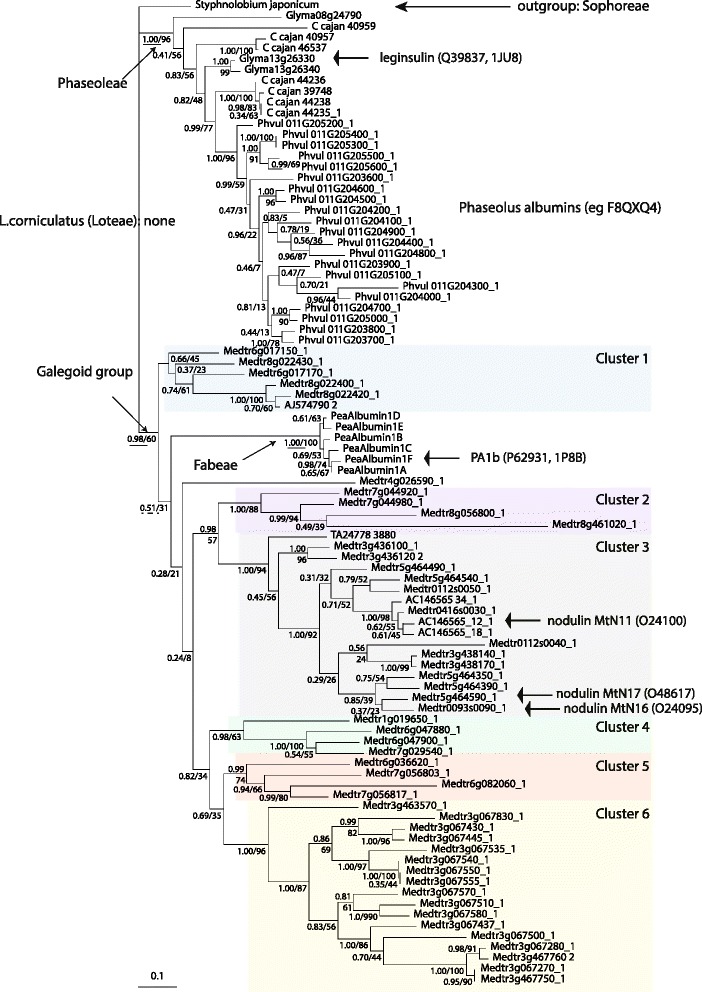
Phylogeny of PA1 homologues identified in the complete genomes of Fabaceae species. Bayesian tree of PA1 homologues identified in *Cajanus cajan* (Phaseoleae), *Glycine max* (Phaseoleae), *Phaseolus vulgaris* (Phaseoleae), *Pisum sativum* (Fabeae), and *Medicago truncatula* (Trifolieae) (91 sequences, 327 nucleotide positions). No homologues were detected in *Lotus corniculatus* (Loteae) and *Trifolium pratense (*Fabeae*)*, and only one in *Cicer arietinum* (Fabeae), which was not included in this tree (see text and Additional file 6: Table S1). The tree was rooted with a sequence from *Styphnolobium japonicum* (Sophoreae), according to the current phylogeny of Papilionoideae [45, 47]. Numbers at branch correspond to posterior probabilities calculated with MrBayes and bootstrap values estimated by PhyML. The *scale bar* represents the average number of substitutions per site. The six *Medicago truncatula* sequence clusters identified previously (Fig. 3) are recovered and indicated with the same colors. Labels on the left identify sustained nodes in the phylogeny of legumes, and labels on the right identify the protein species with substantial experimental data (protein names, Uniprot identifiers & references therein, and PDB identifiers when available). Pea proteins are included as reference peptides (*P. sativum* genome not available yet)

The Bayesian (BI) and Maximum Likelihood (ML) unrooted phylogenic trees of the 53 *Mtr* nucleotide sequences of PA1 were consistent and revealed six well-supported clusters labeled 1–6 (Posterior probabilities (PP) ≥0.98 and Bootstrap Values (BV) ≥75 %, Fig. 3). The analysis of protein sequences provided similar results (not shown). Because these trees contained only *Mtr* sequences, it was not possible to determine if the duplication events, which led to the expansion of PA1 in *M. truncatula,* occurred specifically in this lineage or if they were more ancient within Papilionoidae (the only of the three basal clades of Fabaceae for which A1b sequences are available [18]). To address this question, we searched for homologues in other representatives of the Fabaceae for which genomic data were available. This survey yielded 38 additional A1b sequences from different Papilionoidae: 7 from *Cajanus cajan* (Phaseoleae), 3 from *Glycine max* (Phaseoleae), 21 from *Phaseolus vulgaris* (Phaseoleae), and 6 from *Pisum sativum* (Fabeae). Interestingly, while no A1b sequence was detected in the genome of *Lotus japonicus* (Loteae) and *Trifolium pratense* (Trifolieae), and only one in that of *Cicer arietinum* (Cicereae), a toxic A1b sequence from the Sophoreae *Styphnolobium japonicum*, characterized by homologous PCR [18], and not yet published (C. Royer pers. comm.), was included in the analysis; the *Cicer arietinum* sequence was not included in the phylogeny due to the uncertainty on genome coverage [25]. The BI and ML trees of the 97 PA1 nucleotide sequences were consistent but less resolved than those based on Mtr sequences only due to the more restricted number of positions that could be kept for the analysis. However, they were consistent with the currently accepted systematics of Papilionoidae [30] (Fig. 4). More precisely, A1b sequences from Phaseoleae (*Glycine max*, *Cajanus cajan* and *Phaseolus vulgaris*) formed a separate cluster (PP = 1.00 and BV = 96 %), whereas *Pisum sativum* and *Medicago truncatula* sequences grouped together (PP = 0.98 and BV = 60 %). Within Phaseoleae, the 21 sequences from *Phaseolus vulgaris* formed a monophyletic group (PP = 1.00 and BV = 96 %), indicating a specific expansion of PA1 in this lineage likely through successive duplication events. In contrast, the relationships among the multiple copies of PA1 observed in *Glycine max* and *Cajanus cajan* were not significantly supported (most PP <0.95 and BV <80 %), precluding any conclusion about the wealth of gene duplication events in these two Phaseoleae lineages. Regarding *Medicago truncatula*, the six clusters identified previously were recovered, and all but Cluster 1 were again well supported (Fig. 4). The *Pisum sativum* toxins formed a robust monophyletic group (PP = 1.00 and BV = 100 %) related to *Mtr* clusters (PP = 0.98 and BV = 60 %), their exact relationships with the *Mtr* clusters were not significantly supported. The analysis of protein sequences provided similar trees (not shown).

Altogether, these two phylogenetic analyses suggested that in *Medicago truncatula* i) cluster 1 (laying on chromosomes 6 and 8) could have emerged from the legume toxin ancestor, ii) A1b-nodulins formed a distinct group (cluster 3) laying on two distinctive regions of chromosomes 3 and 5 (*ca.* 3g438000 and 5g464000), iii) the massive expansion of A1b on chromosome 3 resulted from specific successive duplications, and iv) according to functional data available from the literature and from this study (see below), the phylogeny of A1b homologues suggested the toxin activity was ancestral in the *M. truncatula* lineage, and that non toxin A1b (e.g. nodulins) emerged secondarily during the diversification of this gene family, v) the soybean leginsulin lies in an unresolved cluster composed of *Glycine* and *Cajanus* sequences.

### Bioactivity of synthetized peptides

Starting from our phylogenetic analysis and taking into account the molecular requirements for bioactivity [31], we selected and chemically synthesized eight peptide sequences, including the reference molecule pea albumin 1b. We selected two sequences belonging to Cluster 1 (Medtr6g017150 and Medtr017170 referred to as AS37 and DS37, respectively). These were selected for their canonical CRC (R72 in AS37) *vs* non-canonical CYC motif (DS37). In addition, we selected several isoforms (TA24778_3880 and Medtr3g436100 referred to as AG41 and EG41, respectively in cluster 2; AC146565_12 referred to as GL44 in cluster 3; Medtr7g056817 referred to as QT41 in cluster 5 and Medtr3g067510 referred to as AS40 in cluster 6) scattered all over the whole tree but bearing the canonical CRC pattern (Fig. 3 and Additional file 3: Table S3).

The activity of the peptides was assessed for their affinities for the PA1b-binding site, then for their CL50 (lethal concentration 50 %) on cultured *Sf9* insect cells [32]. The peptide activities are reported in Table 1, showing clearly that some sequences did not display toxicity, while others exhibited higher toxicity. More precisely, DS37, QT41 and GL44 peptides did not present binding and toxic abilities while AS37, AG41, EG41 and AS40 sequences revealed binding properties and toxicity. Four peptides (AS37, AG41, QT41 and DS37) were folded with sufficient efficiency to yield the mg amounts needed for a standard *Sitophilus* mortality assay [17], and showed the expected toxicity (AG41 highly toxic, AS37 toxic and QT41/DS37 not toxic; data not shown). Interestingly, the presence of a tyrosine (Y) instead of the canonical arginine R in the CXC motif in DS37 was correlated to an absence of binding and toxic properties. Sequence AS37 led to biological properties similar to those of pea sequences. The AG41 sequence displayed a very high toxicity, almost ten times superior to that of the original pea albumin 1b (Table 1). The toxicity results performed on *Sf9* cultured cells confirmed those obtained by binding affinity, with an overall good correlation between the two assays.

**Table 1:**
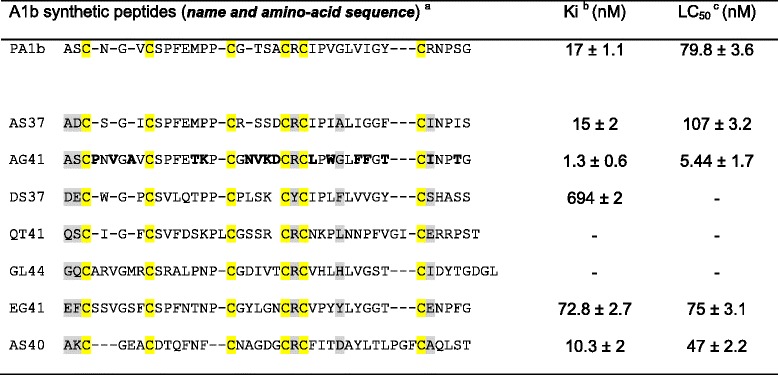
Affinity to the PA1b binding site and insect cell toxicity of synthetic peptides

(−) scores a negative result (no toxicity nor binding in the toxin range assayed)

PA1b the referent molecule and AG41, the Mtr A1b with the highest toxicity were respectively highlighted in grey and pink. The nomenclature for the Mtr A1b (AS37…) was arbitrarily defined as the first and last amino acid in the sequence and the total length. Cysteine architecture is highlighted in yellow. Sites under positive selection in their respective branches are reported as grey background (see PAML analysis, Table 2)

^a^Variant amino-acids in AG41 compared to PA1b sequence are boldfaced

^b^The *Ki* of PA1b and the synthetic peptides was determined by ligand binding using 125I-PA1b, according to [12]

^c^LC50 calculated from biological assays performed on cultured Sf9 cells according to Rahioui et al. [32]

### AG41 acts as a potent blocker of insect cell membrane current

**Fig. 5:**
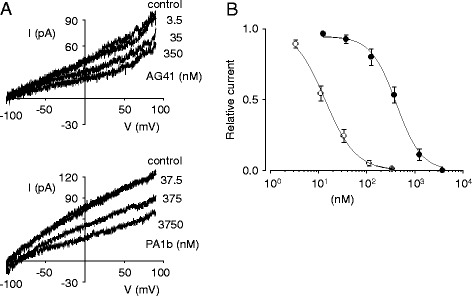
Electrophysiology of two A1b isoforms (PA1b-F and AG41) on insect Sf9 cells. Concentration dependent blockage of Sf9 cells ramp membrane current by AG41 and PA1b. **a** membrane currents recorded in response to voltage ramps of 1.5 s duration applied from −100 to 90 mV in the absence (control) and presence of increasing concentrations of AG41 (*upper traces*) and PA1b (*lower traces*). **b** mean concentration-response data for AG41 (○) and PA1b (●) inhibition of ramp membrane current, measured at +50 mV. Each point represents the mean ± S.E. of *n* = 8–9 independent experiments. Hill equations were fitted to the data, with 100 % blockage taken as the fixed maximum effect, yielding EC_50_ values of 14.6 ± 0.4 nM (*n* = 9, Hill coefficient 1.4 ± 0.1) and 415.3 ± 75.6 nM (*n* = 8, Hill coefficient 1.7 ± 0.2) for AG41 and PA1b, respectively

We further investigated the AG41’s biological activity by performing an electrophysiological experiment on cultured *Sf9* cells to check whether its cell-membrane ion transport alteration differed from that of PA1b, the model pea peptide [13]. Figure 5 shows effects of increasing concentrations of AG41 (upper traces) and PA1b (lower traces) on *Sf*9 cell membrane current recorded in response to 1.5 s duration voltage ramps applied from −100 to 90 mV. At 35 nM, AG41 decreased by approximately 35 % the ramp current amplitude measured at +50 mV (from 68 pA to 44 pA), while a similar concentration of 37.5 nM PA1b had no effect (trace from AG41 even differed from control at 3.5 nM). The concentration/effect relationship was fitted to a Hill equation and yielded EC_50_ values of 14.6 ± 0.4 nM (*n* = 9, Hill coefficient 1.4 ± 0.1) and 415.3 ± 75.6 nM (*n* = 8, Hill coefficient 1.7 ± 0.2) for AG41 and PA1b, respectively (Fig. 5). This experiment essentially showed that AG41 was much more efficient than PA1b to block ramp membrane current in *Sf*9 cells (Fig. 5a), and the Hill number was not significantly different between the two molecules/assays (*p* = 0.14, Wilcoxon test).

### Molecular modeling of AG41 (isoform MtrA1b-013)

**Fig. 6:**
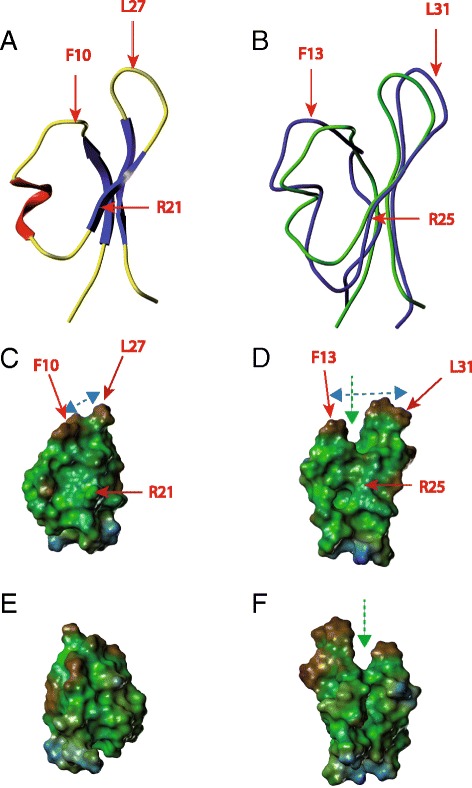
Structures of two A1b isoforms (PA1b-F and AG41). **a** Ribbon representation of PA1b (PDB code: 1P8b). **b** Superposition of the backbones of PA1b (*green*) and AG41 (*blue*). **c**, **d**, **e**, **f** Lipophilic potentials calculated with the MOLCAD option of SYBYL at the Connolly surfaces of (**c**) PA1b and (**d**) AG41. Figures (**c** and **d**) are the same orientation as Figures (**a** and **b**), using a common hydrophobic scale. Hydrophobic and hydrophilic areas are displayed in *brown and blue*, respectively. *Green* surfaces represent an intermediate hydrophobicity. A 180 ° rotation according with respect to a vertical axis is applied from the upper (**c** and **d**) figures to the lower (**e** and **f**) figures

The 3D structure model of AG41 protein (Table 1) was built according to the procedure described in the materials and methods section. As expected, the modeled 3D structure of AG41 adopted the knottin fold typical of PA1b [14] (Fig. 6b). To extend our comparison, we calculated the lipophilic potentials at the Connolly surfaces of the proteins. The 3-D structure delivers these molecular features which are correlated to protein functions. The representation of the hydrophobic properties at the molecular surface of AG41 was typical of an amphipathic structure (Fig. 6c, e). Indeed, AG41 surface displayed a large hydrophobic face formed by the residues of the hydrophobic loop L2: Val25, Leu27, Val28, and Ile29 but also by the facing residue Phe10 of L1. At the other pole of the molecule, the N and C-termini and a part of L1 defined the hydrophilic face with the polar residues Ser2, Asn4, Thr17, Ser18, Asn34, and Ser36. When hydrophobic potentials were calculated using the same hydrophobic scale, the surface of AG41 (Fig. 6d, f) appeared mainly hydrophobic, with a hydrophobic face significantly larger than that of PA1b (Fig. 6d, blue arrows), mainly due to the bulky aromatic residues Trp29, Phe32, Phe33 that were only present in AG41 sequence (Table 1). The enhanced hydrophobicity of AG41, together with the presence of a marked slit at the hydrophobic pole (Fig. 6d, green arrows), could correlate with its increase insecticidal activities, since at least the hydrophobicity of the pole was pointed as a crucial determinant of insecticidal activity [31].

### Selective pressures on *Medicago truncatula* Albumin I sequences

An analysis of the selective pressures was conducted with the PAML package on each of the six identified clusters in *Medicago Truncatula*. Branch models did not allow for the identification of branches with a significantly different evolutionary rate over the whole protein (Table 2). However, site and branch-site models enabled us to confirm previously identified sites under positive selection and even to identify some new.

**Table 2:** Results of selection footprints analysis (PAML site, branch, and branch-site models). Clusters are defined in the general *Medicago*-only phylogenetic analysis described in Fig. 3. A further subdivision of cluster 3 and 6 into two internal sub-clusters (denoted a and b) was defined for site models tests. Branch tested are colour-coded in red in Fig. 3. In each table cell are reported the significance of the model comparison (*p* value), position and ω values of the amino-acid found to be under positive selection in the ‘site’ and ‘branch-site’ analyses after manual curation (see Additional file 4: Table S4 for global alignment positioning)

Cluster	Site model ^(a)^		Branch model ^(b)^	Branch-site model ^(b)^	
cluster_1	*p* = 1.4 10^−4^		ns ^(c)^	ns	
	pos = 83	ω =3.02 +/− 0.78			
	pos = 179	ω =2.97 +/− 0.83			
cluster_2	no ^(c)^		- ^(c)^	-	
cluster_3	*p* = 1.99 10^−13^		ns	*p* = 1.02 10^−4^	
	pos = 27	ω =3.27 +/− 0.59		pos = 74	ω =19.02
				pos = 82	ω =19.02
cluster_3a	*p* = 1.01 10^−3^				
cluster_3b	*p* = 1.24 10^−6^				
	pos = 27	ω =5.54 +/− 1.89			
	pos = 82	ω =5.66 +/− 1.79			
cluster_4	ns		-	-	
cluster_5	no		-	-	
cluster_6	*p* = 1.09 10^−44^		ns	*p* = 6.07 10^−14^	
	pos = 43	ω =8.42 +/− 0.95		pos = 43	ω =19.23
	pos = 76	ω =8.39 +/− 1.07		pos = 92	ω =19.23
	pos = 120	ω =8.42 +/− 0.95		pos = 128	ω =19.23
				pos = 183	ω =19.23
cluster_6a	*p* = 8.04 10^−10^				
	pos = 43	ω =7.074 +/− 1.62			
cluster_6b	*p* = 5.13 10^−15^				
	pos = 92	ω =10.11 +/− 1.25			
	pos = 94	ω =10.20 +/− 0.86			

^(a)^ Probability associated with the LRT between the model M8 and the model M8a

^(b)^ Probability associated with the LRT between the model for which branches in red are considered as foreground branches and the null model (cf. Fig. 3 for branch partition and Method section for models details)

^(c)^ *ns* not significant, *no* no sites validated after manual curation, - no partition tested

Clusters 2, 4 and 5 (the smallest clades that suffer from a lack of statistical power) did not show any signature of positive selection. By contrast, in the deeply-branching Cluster 1, the site model identified two sites under positive selection (see Additional file 2: Table S5 for site numbering), site 83 falling within one of the three critical “spots” for insecticidal activity [31], and site 179 located near the C-terminal ending of PA1a. It is worth mentioning that site 83 was located in the important, and exposed, hydrophobic loop of PA1b and that a significant substitution was present at this site (A- > F) for the two tested isoforms AS37 and DS37. This correlated well with the loss of insecticidal activity following the change to a bulky residue (Table 1). Furthermore, in all insecticidal toxins tested so far, the 180 (L) residue was critical for the insecticidal activity [31] and its neighboring 179 residue was a conserved glycine (tiny) residue. Sterical/hydrophobic constraints also seem to be crucial at that position. The most curious feature in this cluster was, therefore, the absence of almost any trace of expression.

In Cluster 3, position 27 was a significant point of positive selection. It did not lie on the A1b/toxin part of the protein, but rather at the precise position of the signal peptide intron. This gathered the so-called A1b-nodulins, *i.e.* showing nodule-induced expression [33, 34]; changes in the regulatory parts of the gene, including the gene’s canonical intron. The other site under positive selection was at position 82, again in the exposed loop (see Fig. 6), which was no longer hydrophobic within the whole nodulin group. This corresponded to the loss of insecticidal activity observed in GL44 and contrasted with the basal conservation of this activity in isoforms AG41 and EG41 (hydrophobic loop conserved). The last detected site in branch-site analysis within this cluster is at position 74, a site almost adjacent to the critical CXC site located at positions (75–77). The charge distribution within this central (almost buried) site seemed to be an essential component of its activity. In fact, in this cluster, there seemed to be a correlation between charges/residues at positions 74/76, possibly reminiscent of divergent sub-functionalization pressures on the nodulins and their signaling properties.

**Fig. 7:**
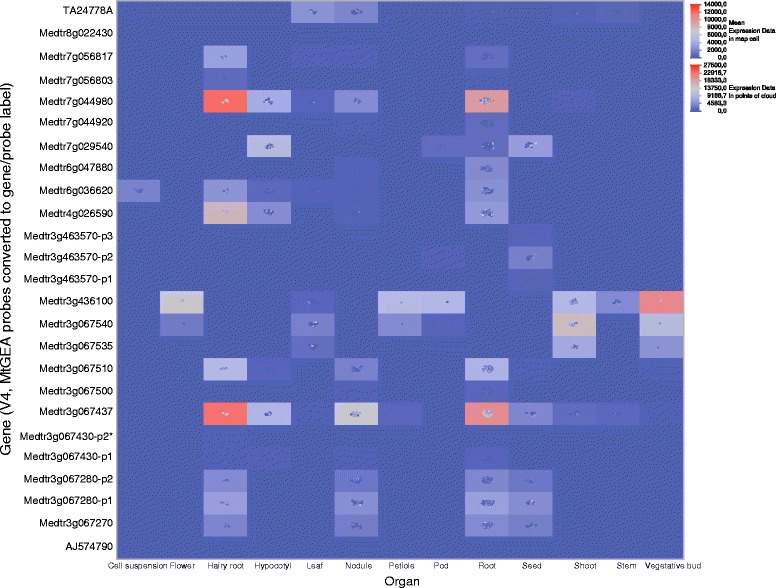
Heat map of all micro-array data available at Mt-Gene Expression Atlas. All (20) A1 genes available in arrays were mapped against their tissue expression, and displayed as a heat-colored cells (mean tissue expression) superimposed with their individual data cloud (showing experiment availability for each tissue –relatively few for flower, petiole *etc.* but many for root, nodule *etc.*–). Original probeset data (Y-axis) were mapped to their corresponding gene in the V4 assembly; three genes are represented by more than one probeset (p1-3; the * star points to a non-100 % match between the corresponding probeset and the V4 genome −92 % nucleic match-). Full Gene/probeset-ID mapping is reported in Additional file 6: Table S1

Finally, the largest and late emerging cluster 6 (chromosome 3 tandem-repeat expansion) also has many sites which seemed to be subjected to positive selection: positions 120, 128 and 183 fall within three otherwise-conserved regions of the PA1a moiety (W128 and K/R128 fall within the HMM-motif defining the Albumin I family in PFAM). Position 43 pinpoints the N-terminus of the A1b peptide in one of the two sub-clusters, while position 92–94 marks the surrounding residues of A1b’s last cysteine, in the other sub-cluster. Both positions were in the hydrophilic part of the molecule, which was not implicated in the insecticidal activity. Finally, the most striking feature of positive selection was residue 76, encompassing all of cluster 6. This residue is located in the hyper-conserved CXC motif, for which an arginine residue is crucial for insecticidal activity (Fig. 6). In the whole cluster, the ratio of the non-synonymous on the synonymous substitutions at this position gives a clear signal of positive selection, which may result from an on-going process of neo-functionalization. Consistent with this interpretation, variations in expression patterns (Figs. 3 and 7) were a clear characteristic of this peptide group. Interestingly, two sequences only retained the large-positive residue at this position (R and Q), one of which confirms its insecticidal activity (AS40, Fig. 3). Whether this was a reversion to, or a conservation of, the ancestral feature requires further analysis.

### Expression analysis of the A1 gene family

#### EST data

*Medicago* expressed sequence tag repositories were carefully searched for all 53 *Mtr* A1 genes. A summary of the results are presented in Additional file 4: Table S4 and mapped on Fig. 3.

The number of ESTs forming each TA varied from 2 to 108 (Additional file 4: Table S4). All these ESTs were classified according to their expression in leaves, roots, seed or others. Almost all A1 genes were expressed in roots (including nodules) (50 % of all ESTs). Roots seemed thus to be a privileged site for the expression of this molecule family in *Medicago*. In contrast, seed expression was exceptional (4 % of all ESTs). In addition, 16 % were expressed in leaves and 28 % were found in other tissues/organs. Some genes (Medtr 3g067430, cluster 6; Medtr 3g436100, cluster 3) exhibited ubiquitous expression while others were organ-specific (Medtr3g463570, cluster 6, in seeds). Among the five genes expressed in seeds, the diversity of the CXC motif was of particular interest, where the typical insecticidal arginine residue was replaced by I, E or F suggesting the loss of insect-toxic abilities of theses peptides.

#### Microarray data

Extensive micro-array data for the species *Medicago truncatula* were available at the gene expression atlas site MtGEA (http://mtgea.noble.org/v3/). We retrieved all data concerning the available A1b genes, resulting in a 25 × 920 table (25 probe sets representing 20 different genes; 920 modalities from *ca*. 43 experiments). The experiments were classified with a 13-tissue classification scheme and all data were mapped on a mixed cloud/heat-map outline, as shown in Fig. 7. The correlation between EST and micro-array data was generally good, although some discrepancies were noted (5g464350, 5g464590 & AC146565_12 were identified in root-expressed tags but were not present on microarrays; Medtr3g067430 was ubiquitously present in the EST data but almost undetectable in the micro-array data). Statistical artifacts arose with low-populated map cells (hairy root, flower or vegetative buds), but major global features appeared, confirming the higher root expression (a mean 20–25 x expression in roots/hairy roots as compared to stems or cell suspensions; a two-way gene x organ Anova was performed, not shown). Multi-probe genes showed coherent patterns. Also, some expression correlations appeared between closely associated genes, with notable exceptions: Medtr7g044920 and Medtr7g044980 displayed very distinct profiles, and Medtr3g067437 could be easily differentiated from its neighbors. The two latter genes (roots) were, together with Medtr3g436100 (vegetative buds) the highest expressors (Fig. 7). Interestingly, Medtr7g044980 lay on the shorter branch of Cluster 2 and should not be insecticidal, as is the case with Medtr3g067437, which lay in an isolated long branch of Cluster 6, while Medtr3g436100 encoded an insecticidal peptide, and was branched basally in Cluster 3 (EG41 isoform). The EST expression of the latter gene confirmed a large tissue distribution but with a low root-expression. The main three A1b expressors were therefore likely to display very different functions in *M. truncatula* and only one of them retained its ancestral insecticidal function. A1b-nodulins were not plotted in our heat-map (not all were retrieved by the Albumin I keyword) and they were low/conditional-expressors, which concurred with their proposed signaling functions.

Finally, grouping genes according to their similar tissue-expression profiles resulted in the following organization (Fig. 7): conductive organs (stem, shoot, petiole, buds, pods) and flowers showed a quite similar expression of three genes among which Medtr3g436100 (EG41, insecticidal) seemed to be paradigmatic. Seed expressors, such as Medtr7g029540 (Cluster 4) or a couple of Medtr3g067nn genes (Cluster 6), all encoded peptides lacking the crucial positively charged bulky residue (R, eventually K) necessary for insecticidal activity. Seed expressors will also be highlighted in the next (proteomic) section. Root expressors were more numerous and usually not predicted to be insecticidal, with the noticeable exception of medtr3g067510 (AS40). Nodule expressors were, in general, like root expressors, with again the noticeable exception of the orphan expressed tag TA24778, which was also one of the few A1b isoforms expressed in the leaf. Its high insecticidal activity (see AG41 later) was associated with this atypical expression profile (although it was also found at low levels in root ESTs).

#### Proteomic data

Previous data on *Mtr* seed proteomics [17, 18] failed to reveal any expression of the two genes first identified from this species. Our present data clarified these results in that both genes identified by homology (genomic PCR; Medtr8g022430 and AJ574790) have been shown to be silent or only expressed at very low levels, although quite conserved (Fig. 3, Cluster 1). On the other hand, our raw proteomic data were reanalyzed with the transcriptomic and genomic data now available (Additional file 5: Table S8) and this resulted in the detection, in the seed extracts, of most of the A1b isoforms identified as seed expressors. The hydrophobic peptide fraction, usually containing standard legume albumins A1b contained only traces of expression of Medtr3g067540 [17], while the polar peptide fractions [18] contained the newly identified peptides from V4 of the *Mtr* assembly, distributed between the acidic polar fraction (genes, Medtr3g067270 cluster 6 and Medtr3g067540 cluster6) and the basic polar fraction (genes Medtr3g067270 cluster 6, Medtr3g067430 cluster 6, Medtr3g067540 cluster 6, Medtr3g436100 cluster 2 and/or Medtr3g067280 cluster 6 and Medtr3g463570 cluster 6).

## Discussion

### Albumins I are more diverse in *Medicago truncatula* than in any other legume

*Mtr*-A1 peptides (Fig. 1) are encoded by a multigenic family, currently comprising 53 members distributed along all but one of the eight *Medicago truncatula* chromosomes. Although previously unsuspected for A1bs, this complexity seems to result from a lineage-specific gene expansion, maybe starting from the loci on chromosome 6 or 8, through successive rounds of duplications. In plant genomes, gene enrichment through duplication is commonplace, as occurs with the ERF transcription-factors in cucumber [35], and is mainly due to polyploidization, segmental and tandem duplications. An example is the non-specific lipid transfer protein (ns-LTP) in wheat *vs* rice and *Arabidopsis* [36]. Recently, such mechanisms were also found to be implicated in domestication processes [37]. Tandem duplication events occurred repeatedly on chromosomes 3 and 5 (Figs. 2 and 3). The pattern of duplications also points to evolutionary links among sequences that lay on different chromosomes: chromosomes 6 and 8 (cluster 1), chromosomes 6 and 7 (cluster 3), and chromosomes 7 and 8 (cluster 5). The expression pattern and selective pressure analyses shows that a number of sub- and neo-functionalization processes had occurred during the diversification of A1bs in *M. truncatula*. This is not surprising for a single small molecule that has already been implicated in three independent biological functions/targets, namely insect-plant interactions, plant signaling/phosphorylation [38–41], and regulation of glycaemia in mammals [42].

### No parent peptide family was detected in other plant lineages

Until now, the A1 gene family has been restricted to legume plants. This concurs with A1bs being the only cysteine-rich peptide family, among more than 30 other known families, which was not present in any of the *Arabidopsis* and rice genomes [19]. Interestingly, the genus *Clitoria* (Fabaceae: Phaseoleae) was recently shown to harbor a novel type of cyclotides, which was identified as a chimeric assemblage of a C-terminal PA1a and an N-terminal cyclotide [43, 44]. However, the evolutionary history of such chimeric molecules is not known but it strongly suggested that a recombination event has affected the PA1 coding gene present in the ancestor of Phaseoleae, leading to the replacement of the PA1b domain by a cyclotide domain in the Clitoria lineage. Another intriguing situation is the recent identification of an A1b gene acquired by horizontal gene transfer by *Phelipanche aegyptiaca* and related species (Lamiales, Orobanchaceae), not included in our dataset, which has likely conserved the insecticidal function [20]. All these examples illustrate both the recombination properties of the two A1 functional modules (A1a and A1b) and the evolutionary stability of their respective signatures with the ability to persist in recipient organisms or genomic contexts long after the recombination event.

### The insect toxin function seems to be ancestral in legume A1b evolution

The phylogenetic analysis of the PA1 family strongly suggested that the original legume gene present in the ancestor of the studied Papilonoideae coded for a toxin aiming at protecting seed from insects. *S. japonicum* is the most ancient and basal Papilionoideae species for which experimental data of the presence of A1b peptides is available, initially through mass spectrometry and biological data [18], and now through sequence information. The Cladrastis clade, to which *S. japonicum* belongs, is a basal legume tree group lying close to the Swartzieae and ADA clades, at the base of the Papilionoidae [45], and a sister group to the so-called 50 kb-inversion group (plastid genome rearrangement) comprising most of the common Papilionoidae. Therefore, *S. japonicum* (syn. *Sophora japonica*, the Japanese pagoda tree) harbors the more divergent albumin I known to date. Consensus legume phylogeny therefore dates the A1b family back to the ancestor of *S. japonicum* and all other known families harbouring such peptides, especially the genistoid clade –genus *Lupinus*; Uniprot Q96474 [46], with an estimated 56–58.5 My/late paleocene origin [47].

Remarkably, multiple expansions of the PA1 family occurred during the diversification of Phaseoleae and Fabeae through successive gene duplications. While, in some species (e.g. *Pisum sativum* [16, 17] and *Phaseolus vulgaris* [48, 49]) the resulting paralogues have kept the ancestral insecticidal function, in *Medicago truncatula* and in *Glycine max* functional diversification occurred (high in *Mtr*, low in *Gma*). It may be noted that the insect-toxin itself might not be mono-functional: the insecticidal homologue of PA1b in *G. max* (Glyma13g26330 Fig. 4), also named leginsulin, was first studied for its hormonal and signaling functions in soybean seeds [38–41].

### Diversified A1b expression and function in *Medicago*: no longer a seed toxin

One of the most striking results of this genomic survey was that the standard situation prevailing in all other legumes studied so far, namely that albumins are toxic seed storage proteins, is not true in *Medicago truncatula*. Instead, most genes that showed seed expression (transcripts or peptides) are predicted not to be insect toxins, with the exception of EG41/Medtr3g436100 cluster 3. Moreover, we were unable to detect toxic peptides in *M. truncatula* seeds using our original homology-based genomic PCR strategy [17], which was leading to the cluster_1 members that had lost their expression. In relation to their shift of expression out-of-seeds, *Medicago* A1bs acquired an extremely diversified tissue distribution, dominated by root expression, which accounts for the presence of almost half of the array-detected transcripts (Fig. 7). This emerging pattern seems an alternation of loss/gain and conservation of function, linked to expression retargeting. The current positive selection traces on two of the “functional hot spot” of the molecule [31] is a clear indication of occurrence of discrete stepwise functional changes. However, this assumption needs to be checked experimentally by functional studies.

### Nodulin and chromosome 3 expansion as recent neo-functionalization bursts

Nodulins were defined by substractive expression methodologies as genes specifically expressed in legume rhizobial-associated nodules [34, 50]. Early in this process, and after the identification of many transport-associated membrane proteins, some small proteins involved in signaling were identified (ENOD peptides, for Early-NODulins) [34]. In this line of research, three nodulins MtN11 (AC146565 cluster3), 16 (Medtr0093s0090 cluster 3) and 17 (Medtr5g464590 cluster3), were identified [34] and these happened to be distant and short homologs of the albumins 1, but their functions were not investigated further. Our work unveiled a group of 14 homologues to the first three described (Fig. 3), that are all short and mostly 6-cysteine peptides (A1b only, see alignment in Additional file 2: Table S5). They are grouped in Cluster 3 and are basally related to a set of three more canonical sequences, two of which being readily insecticidal (AG41, EG41). The so-called “nodulin” cluster is therefore arising from apparently standard toxins that have lost their PA1a moiety and have subsequently undergone significant sequence changes albeit retaining the cysteine scaffold (with the exception of 3 very-short members). Traces of selection were detected at the basal branch of each of the nodulin sub-groups, namely the isolated outgroup Medtr5g464490 and the two sub-clusters 3a and 3b defined for the PAML analysis. In addition to the site features discussed in the results section, it is interesting to note that the loss of the PA1a domain is correlated with significant sequence changes, including that of the canonical hydrophobic loop and a total loss of insecticidal activity, even when the canonical CRC stretch is retained (MtN11 = GL44, Fig. 3 and Additional file 2: Table S5). In this putative “nodulin” cluster, it is likely that the structural constraints for knotted peptide folding are released, which would fit with the absence of a PA1a moiety, as this domain is now strongly suspected of serving as a chaperone for assisted co-translational folding of most canonical PA1b peptides [51]. Since knottins are not all difficult to fold [52], the presence of a flanking pro-peptide chaperone is probably useful in a restricted part of the conformational space explored by the A1bs from *Medicago truncatula*. Most cyclotides do not need chaperones either [53].

### Insect toxins after all: one extinct and two active clusters?

The final issue deals with the expression of insect toxicity in *Medicago truncatula* tissues. We are well aware that our experimental data (7/53) is extremely partial, but we are now confident that the predictive power concerning insecticidal activity from sequence information is relatively good. The first question pertains to Cluster 1 genes, for which the expression data was almost non-existent. The data was checked for two genes and it was confirmed with NGS data; genes Medtr6g017150 (AS37) and Medtr6g017170 (DS37) revealed no expression whatsoever (Pascal Gamas, pers. comm.). As a control, we also checked two other EST-orphan genes from the “nodulin” cluster: they were shown to be expressed, and induced, in nodules (Medtr5g464350 being significantly expressed, while a Medtr3g438170-like signal was very weakly observed in nodules too). We are therefore confident that Cluster 1 is globally composed of silent genes, although one of them (AS37) conserved its insecticidal activity.

Apart from this silent gene set, our study detected two other clusters with interesting insecticidal activities: the nodulin-related cluster 3 (Figs. 3 and 7, AG41 and EG41) and the only CRC-containing member from the chromosome 3 expansion cluster 6. These two groups are expressed in roots and nodules, but AG41 shows an interesting and strong conditional expression in nodules (Fig. 7), as well as a good expression in leaves. From an ecological point of view, expressing a very potent insecticidal molecule in a high-value nitrogen source organ would not be fortuitous, as suggested by the fact that the nodule-specific (adapted) insects seem devoid of receptor binding sites, and therefore susceptibility to A1-type toxins [7, 11].

## Conclusions

When viewing our survey as a search for protein innovation within a specific taxon (namely legumes), one may ask how unusual are A1b in this respect ? A screen of the Interpro [54] and Pfam [55] databases for taxonomic boundaries of protein domain families retrieved only three families that are restricted to (and were therefore invented in) the Fabaceae/legumes: *Albumins 1*, *Nodule-specific glycine rich proteins*, and the *late nodulin family*. One may add the NCR expansion to this list [56, 57], which is not captured by a single protein family, and is distinct from A1 (for example by cysteine topologies). Many other nodule-specific proteins were subsequently recovered in other plant taxa, such as *enod* hormones, and even the typically nodular leghemoglobin, related to the very ancient heme-binding globin family. Not surprisingly, these major protein novelties are somehow related to rhizobial symbiosis (A1s via nodulins), but also concern small proteins. This may illustrate how protein modules can be derived by a process of neofunctionalization of a previously existing scaffold (e.g. nodulins from the ancient insecticidal A1b group, maybe derived itself from a still undiscovered cysteine-rich family).

In conclusion, the study of the multigenic insecticidal albumin1 family is of interest to the evolutionary history of legume-specific protein families, and to novel bioactive molecule discovery. The exploration of our results may end up in new biopesticide leads, such as AG41, for the control of a large array of insect pests [7].

## Methods

### Identification of A1 genes in available genomes of *Fabaceae*

Our work was initially based on version Mtr_3.5_v5 of the *Medicago truncatula* genome assembly and translation (http://www.jcvi.org/cgi-bin/medicago/download.cgi), and it was further extended to version 4, kindly provided, upon request, by the JCVI team on march 15 2013 (Mt4RC1_ProteinSeq_20130326_1624.fasta). Blastp [58] was first run on the official protein sets of *Medicago truncatula* (e.g. on file Mt4RC1_ProteinSeq_20130326_1624.fasta of the 84,993 proteins of v 4) with the two seed sequences corresponding to published pea and barrel medic albumins 1 (Uniprot P62931 and G7L8D8), with default settings. This retrieved a set of 47 coding sequences displaying the canonical penultimate CXC topology of albumins 1, which could be assigned to the albumin 1 family. With this set, a second round of blastp was run, with relaxed parameters, and a set of 50 protein hits was retrieved. Two of them were excluded as being blast false positives (one Cobra-like cysteine-rich protein Medtr3g438140, and one reverse transcriptase zinc-binding protein carrying 6 cysteines, Medtr7g071493). Thus, only one new coding sequence was thus added by the second blast round, indicating that A1 albumins are essentially a unique isolated protein family with very low sequence similarities with other families within the *Medicago truncatula* genome.

The LEGoo platform was also used to acquire information on the retrieved genes from v3.5 (a bioinformatics gateway for integrative legume biology: www.legoo.org). Likewise, the *Cajanus cajan* and *Glycine max* A1s were found in the LEGoo and Genbank databases respectively. The *Lotus japonicus* genome was also searched for A1 sequences in LEGoo but none was found. The *Phaseolus vulgaris* genome was screened with blast on the phytozome website (http://www.phytozome.net). The *Cicer arietum* and *Trifolium pratense* genomes were also searched for A1 sequences through http://cicar.comparative-legumes.org/, http://www.nipgr.res.in/ctdb.html, and http://www.plantgdb.org/but yielded only one hit for chick pea (Additional file 3: Table S3), and were not used for phylogeny. Our species selection scheme is summarized in Additional file 6: Table S1 and attained the six quality assembled genomes used in Fig. 4. Finally, we used all the *Pisum sativum* sequences published in Uniprot at the beginning of 2014. The *Styphnolobium japonicum* sequence (EMBL accession number: LN854577) was obtained by using our original homology-based genomic PCR strategy [17].

Sensitive HMM-based homology searches were performed in an attempt to extend the albumin 1 family. Specific HMM profiles were built from the multiple alignments of ProDom2010.1 families PDA1L0K4 and PD015795 [59] corresponding to albumin A1a and A1b families, respectively. These HMM profiles were compared with all sequences in the UniProt database using HMMER3 [60]. Matches were considered on the basis of an ‘independent’ E-value below 0.01. Alternatively, recursive homology searches were performed using the jackhmmer program with consensus sequences of the same ProDom families as queries and the same independent E-value cut-off.

### *Medicago truncatula* EST database searches

A blastp, using the same seed sequences as for the genomic search, was performed on the *Medicago truncatula* gene index (MtGI, version 11: http://compbio.dfci.harvard.edu) and on the TIGR plant transcript assemblies (http://plantta.jcvi.org). The same two-step blast strategy was performed as for the genomic searches and this retrieved 50 transcript families that were checked for false positives as previously described. Cobra-like and PR10 family members were first excluded, as well as 4 NCR (nodule cysteine-rich peptides) that did not display the canonical penultimate CXC topology of albumins. A total of 33 expressed sequences was retained, and was matched to V4 genes by local blast. Only one EST was kept orphan, and all the *Medicago* A1-family unigenes are presented in Additional file 1: Table S2, together with the identification synonyms between databases and assembly versions.

In determining the tissue-specificity of identified transcripts on TIGR and MtGI databases, the following clustering terms were considered: seed(s), leaves, roots and others. The category “others” included: seedlings, plantlets, cotyledon, stems, flower, nodules and isolated glandular trichomes.

### Amino-acid sequence analysis

Pre-pro-proteins, translated from the open reading frame of all A1 sequences, were analyzed for the presence of potential signal peptide cleavage sites using the SignalP 4.0 program [61], and prediction statistics were gathered for further analysis. Following signal peptide removal, theoretical isoelectric points (pI) and molecular weights (MW) of (PA1b + pro-peptide) were computed using Expasy’s pI/Mw tool (http://web.expasy.org/protparam/) [62]. All this information is summarized in Additional file 1.

### Phylogenetic analysis of exons/proteins

The 53 A1 protein sequences of *Medicago* were aligned using MAFFT v7 with the *linsi* option which allows accurate alignment reconstructions [63]. The quality of the alignment was controlled and adjusted manually with SeaView v4.4.2 [5]. A second alignment including the 38 homologues detected in *Pisum sativum, Cajanus cajan, Phaseolus vulgaris* and *Glycine max* was constructed according to the same strategy. Using these two multiple alignments as guide, the corresponding nucleotide sequences were aligned. The regions of protein alignments where the alignment was doubtful were removed with the version of Gblocks implemented in SeaView (less stringent parameters) and manually adjusted. The corresponding regions were removed from the nucleotide alignments. All alignments are given in additional files 7 and 8.

Maximum likelihood and Bayesian trees were inferred with PhyML v3.1 [64] and MrBayes v3.2 [23], respectively. Phylogenetic analyses of nucleotide alignments were performed with the GTR model. A gamma distribution with four categories of sites was included to take into account the heterogeneity of site evolutionary rates (estimated alpha parameter). While maximum likelihood phylogenetic trees of protein sequences were inferred with the Le and Gascuel model [65], the mix model was used for Bayesian inferences. For PhyML, the NNI + SPR option was used for the tree space exploration for the maximum likelihood inference and the robustness of the maximum likelihood trees was assessed with a parametric bootstrap procedure (100 replicates of the original dataset). For MrBayes, four chains were run in parallel for 1,000,000 generations. The first 2000 generations were discarded as burn-in. The remaining trees were sampled every 100 generations to build consensus trees and compute posterior probability.

### Evolutionary rate (PAML) analysis

In order to investigate the selection pressures driving evolution of the albumin family, different models allowing the dN/dS ratio (ω, *i.e.* the non-synonymous on synonymous substitution rate ratio) to vary, were tested using the codeml program of the PAML4 software [66]. Three kinds of models were used: ‘site’ models, where the dN/dS ratio is allowed to vary between sites; ‘branch’ models where the dN/dS ratio is allowed to vary between branches; and ‘branch-site’ models where the dN/dS ratio is allowed to vary between both branches and sites. Tests were implemented in homemade python scripts, relying on the egglib package [67].

Models were tested on clusters 1 to 6 and, for each cluster, the sub-tree topology was maintained as it appears in the general tree (Fig. 3). For the ‘site’ models, the nearly neutral model (M8a) assumes codons do evolve either neutrally or under purifying selection. The positive selection model (M8) assumes that, in addition to codons evolving either neutrally or under purifying selection, a certain proportion of codons are evolving under positive selection (ω >1). Likelihood ratio tests (LRTs) were performed to compare M8 with M8a and, hence, to detect clusters for which models that include positive selection are more likely than models that do not. In clusters identified as having evolved under positive selection, Bayes empirical method was used to calculate the posterior probabilities at each codon and to detect those under positive selection (*i.e.* those with a posterior probability of having a dN/dS >1 above 95 %). Sites detected to be under positive selection at the codon level were curated manually for alignment quality and reliability. We were very stringent since some parts of the protein appeared to evolve extremely quickly. In those parts of the protein, positively selected sites were declared true positive only if they were surrounded by conserved sites and with no indels in the considered cluster’s own alignment.

For the ‘branch’ and ‘branch-site’ models, branch partitions need to be defined *a priori* so we used the method implemented in mapNH [68], which performs substitution mapping on branches. Note that the total number of non-synonymous and synonymous sites per alignment was computed by codeml during the site model analysis. We used this information to define partitions: we selected branches with dN/dS >1.4 and tested if the ‘branch’ model with different dN/dS for these branches was more likely than a model with the same dN/dS in all branches. As multiple testing is implicit in this method, we corrected the *p*-values using the total number of branch partitions that can be tested for each cluster. Note that for branches containing no synonymous or no non-synonymous mutations, or no mutation at all, dN/dS could not be properly computed by mapNH. Thus, those branches were always considered as background branches.

Branch partitions tested with ‘branch-site’ models were the same as for the ‘branch’ models. Branches with dN/dS >1.4 were defined as foreground branches and those with dN/dS <1.4 as background branches. Then two models were compared: the null model (A0), in which sites on the fore- and background branches evolved under the same selective pressure (purifying or neutral), and a model including positive selection (model A) in which some sites on the foreground branches evolved under positive selection whereas sites on the background branches still evolved under purifying selection or neutrality. Again, the most likely model was inferred by LRT and sites detected to be under positive selection at the codon level were curated manually for alignment quality and reliability.

### Structural sequence alignments and comparative modeling

Sequence alignments (Table 1) were performed using CLUSTAL OMEGA1 [69]. Comparative modeling was performed using ORCHESTRAR homology modeling program in the SYBYL-X 2.0 software package (TRIPOS Inc., St Louis, MO). PA1b NMR structure (PDB code: 1P8b) was used as a template to build the 3D structure model of the AG41. Lipophilic potentials were calculated and represented using the MOLCAD option of SYBYL-X 2.0 software package.

### Chemical synthesis, oxidative refolding and peptides purification

The crude peptides of PA1b isoforms were obtained from Proteogenix (Strasbourg, France). They were then folded following the optimized procedure described for the production of synthetic PA1b [70]. The isoforms were purified by semi-preparative RP-HPLC. The purity of the peptides was assessed using RP-HPLC and matrix-assisted laser desorption ionization-time of flight (MALDI-ToF) mass spectrometry. The concentration of the peptide was determined by measuring its RP-HPLC peak area at 210 nm, referring to known quantities of pure peptides used as standards.

### Biological material and toxicity assays

All synthetic peptides have been tested on *Spodoptera frugiperdaSf9* cells lines kindly provided by Martine Cerutti, University of Montpellier, France. *Sf*9 cells lines were grown at 27 °C in Lonza’s culture medium, supplemented with 5 % fetal bovine serum (FBS) and 0.1 % gentamicin. *Sf9* cells were seeded, in 96-well plates, 24 h prior to the experiments (15 000 cells/well) and were exposed to increasing concentrations of synthetic peptide. After this 24 h period, cell viability was determined using the CellTiter-Blue Viability Assay (Promega), according to the manufacturer’s instructions. After addition of the dye, the cells were incubated at 27 °C for 4 h. The absorbance, at 570 and 600 nm, was then measured using a microplate reader (MR 7000, Dynatech Laboratories Inc., USA).

### Binding assay

The affinity of synthetic peptides for the PA1b-binding site was determined by ligand binding using ^125^I-toxin [12].

### Electrophysiology

Current recordings were made at room temperature, under voltage clamp using the whole-cell configuration of the patch-clamp technique. Command voltage and data acquisition were performed with pClamp software (Axon Instruments, Foster city, CA, USA). The holding potential was −80 mV. Membrane currents were evoked, every 10 s, by voltage ramps of 1.5 s in duration applied from −100 to 90 mV, filtered at 300 Hz and sampled at 1 kHz with an analogue-to-digital converter (Labmaster TM 40, Scientific Solutions Inc., Solon OH, USA). The external solution contained (in mM): 135 NaCl, 4 KCl, 2 MgCl_2_, 10 CaCl_2_ and 10 Mes, adjusted to pH 6.4 with NaOH. The internal solution contained (in mM): 140 K-aspartate, 1 MgCl_2_, 3 K_2_-ATP, 5 EGTA and 5 Hepes, adjusted to pH 7.2 with KOH. Salts were purchased from Sigma-Aldrich and peptides were prepared as 60 % (*v/v*) ethanol stock solution as previously described [13].

### Statistics

All statistical analyses and related graphical displays (e.g. heatmap) were performed under JMP V11 software (SAS Institute). Biological results are expressed as the mean ± S.E.M., *n* indicating the number of *Sf9* cells studied (electrophysiology). Statistical comparisons were made using a Mann–Whitney U or Wilcoxon tests at the 95 % confidence level.

### Availability of data and material

The datasets supporting the conclusions of this article are included within the article and its additional files.

## Additional files

Additional file 1: Table S2. PA1 genes identified in the Medicago truncatula genome and features of the deduced proteins. A cluster of tandem duplication repeats is indicated by a vertical line in front of the gene names. AA, number of amino acids; MW, molecular mass in Dalton; pI, isoelectric point; SignalP 4.1 was used to predict the cleaving site of signal peptide; ^a^ EMBL ID of *Pisum sativum;* http://web.expasy.org/compute_pi/ was used to determine the pi and MW values; In the absence of signal peptide cutoff prediction, the complete sequence is shown in Additional file 1: Table S1; ND indicates not determined. (XLSX 16 kb) Additional file 2: Table S5. Position-numbered alignment of all *Medicago truncatula* A1b proteins, grouped and colored by identified clusters/clades (see Fig. 3) (DOCX 87 kb) Additional file 3: Table S3. Diversity of the cysteine motif in *Medicaco truncatula* PA1b types. The consensus motif of each PA1b type was deduced from the analysis of the mature sequences of the 53 *Medicago truncatula* PA1b presented in Additional file 1: Table S1. Medtr3g438140.1 and Medtr3g438170.1 are very short sequences and were excluded from the survey. Similarly, Medtr8g056800 was excluded from this table because it lacks the A1b sequence. A cysteine residue is missing in AJ574790.1 and Medtr3g467750.1. Medtr3g067830.1 and Medtr6g082060.1 subfamily members harbor an extra cysteine residue. Medtr3g067445.1 and Medtr3g067430.1 subfamily members harbor two extra cysteine residues. Medtr6g082060.1 displays no CXC motif. (XLSX 15 kb) Additional file 4: Table S4.
*Medicago truncatula* tissue gene expression data. This data set was based on expression data from the TIGR gene indices. Others are defined in Methods. (XLSX 10 kb) Additional file 5: Table S8. Proteomic data for *Medicago truncatula* seed peptides. Raw analysis of peptides matching from Maldi-Tof analyses, previously published by our group [17, 18]. Reanalysis using the *Mtr* genome V4 assembly. (XLSX 12 kb) Additional file 6: Table S1. Legume species analyzed. Legume species selection data. Genomes with high quality full genomes used for phylogenetic analysis, and additional genomic data sources. (XLSX 82 kb) Additional file 7: SM5-UnigeneAllGenomes[91pep].fasta. Alignment file for all legume Albumin I peptides (corresponding to species from tree in Fig. 4). (FASTA 20 kb) Additional file 8: SM6-UnigeneAllGenomes[91cds].fasta. Alignment file for all legume Albumin I coding sequences (corresponding to phylogenetic tree showed in Fig. 4). (FASTA 50 kb)

## Abbreviations

A1b: albumin 1b peptides
BI: bayesian
BV: bootstrap values
*Car*: *Cicer arietinum*
*Cca*: *Cajanus cajan*
EC50: effective concentration50
ENOD: early-NODulins
*Gma*: *Glycine max*
LC50: lethal concentration 50 %
*Lja*: *Lotus japonicas*
LRTs: likelihood ratio tests
ML: maximum likelihood
*Mtr*: *Medicago truncatula*
NCR: nodule-specific cysteine-rich
PA1: pea albumin 1
PAML: phylogenetic analysis by maximum likelihood
PP: posterior probabilities
*Pvu*: *Phaseolus vulgaris*
*Sf9*: *Spodoptera frugiperda 9*
*Tpr*: *Trifolium pratense*

## References

[1] Graham PH, Vance CP (2003). Legumes: importance and constraints to greater use. Plant Physiol.

[2] Dixon RA, Sumner LW (2003). Legume natural products: understanding and manipulating complex pathways for human and animal health. Plant Physiol.

[3] Duke JA (1992). Handbook of legumes of economic importance.

[4] Southon IW, Bisby FA, Buckingham J, Harborne JB, Zarucchi JL (1994). International Legume Database and Information Service., Chapman & Hall Chemical Database.

[5] Gouy M, Guindon S, Gascuel O (2010). SeaView version 4: A multiplatform graphical user interface for sequence alignment and phylogenetic tree building. Mol Biol Evol.

[6] Wink M (2003). Evolution of secondary metabolites from an ecological and molecular phylogenetic perspective. Phytochemistry.

[7] Rahioui I, Eyraud V, Karaki L, Sasse F, Carre-Pierrat M, Qin A, Zheng MH, Toepfer S, Sivignon C, Royer C (2014). Host range of the potential biopesticide Pea Albumin 1b (PA1b) is limited to insects. Toxicon.

[8] Delobel B, Grenier AM, Gueguen J, Ferrasson E, Mbaiguinam M. Utilisation d’un polypeptide dérivé d’une albumine PA1b de légumineuse comme insecticide. Patent 98/05877 C12N15/29, C07K14/415, A01N65/00. France; 1998

[9] Gressent F, Da Silva P, Eyraud V, Karaki L, Royer C (2011). Pea Albumin 1 subunit b (PA1b), a promising bioinsecticide of plant origin. Toxins (Basel).

[10] Gatehouse JA, Gilroy J, Hoque MS, Croy RR (1985). Purification, properties and amino acid sequence of a low-Mr abundant seed protein from pea (*Pisum sativum* L.). Biochem J.

[11] Gressent F, Duport G, Rahioui I, Pauchet Y, Bolland P, Specty O, Rahbe Y (2007). Biological activity and binding site characteristics of the PA1b entomotoxin on insects from different orders. J Insect Sci.

[12] Gressent F, Rahioui I, Rahbe Y (2003). Characterization of a high-affinity binding site for the pea albumin 1b entomotoxin in the weevil Sitophilus. Eur J Biochem.

[13] Chouabe C, Eyraud V, Da Silva P, Rahioui I, Royer C, Soulage C, Bonvallet R, Huss M, Gressent F (2011). New Mode of Action for a Knottin Protein Bioinsecticide: Pea Albumin 1 Subunit b (PA1b) is the first Peptidic Inhibitor of V-ATPase*. J Biol Chem.

[14] Jouvensal L, Quillien L, Ferrasson E, Rahbe Y, Gueguen J, Vovelle F (2003). PA1b, an insecticidal protein extracted from pea seeds (Pisum sativum): 1H-2-D NMR study and molecular modeling. Biochemistry.

[15] Gelly JC, Gracy J, Kaas Q, Le-Nguyen D, Heitz A, Chiche L (2004). The KNOTTIN website and database: a new information system dedicated to the knottin scaffold. Nucleic Acids Res.

[16] Higgins TJ, Chandler PM, Randall PJ, Spencer D, Beach LR, Blagrove RJ, Kortt AA, Inglis AS (1986). Gene structure, protein structure, and regulation of the synthesis of a sulfur-rich protein in pea seeds. J Biol Chem.

[17] Louis S, Delobel B, Gressent F, Rahioui I, Quillien L, Vallier A, Rahbé Y (2004). Molecular and biological screening for insect-toxic seed albumins from four legume species. Plant Sci.

[18] Louis S, Delobel B, Gressent F, Duport G, Diol O, Rahioui I, Charles H, Rahbe Y (2007). Broad screening of the legume family for variability in seed insecticidal activities and for the occurrence of the A1b-like knottin peptide entomotoxins. Phytochemistry.

[19] Silverstein KA, Moskal WA, Wu HC, Underwood BA, Graham MA, Town CD, VandenBosch KA (2007). Small cysteine-rich peptides resembling antimicrobial peptides have been under-predicted in plants. Plant J.

[20] Zhang Y, Fernandez-Aparicio M, Wafula EK, Das M, Jiao Y, Wickett NJ, Honaas LA, Ralph PE, Wojciechowski MF, Timko MP (2013). Evolution of a horizontally acquired legume gene, albumin 1, in the parasitic plant Phelipanche aegyptiaca and related species. BMC Evol Biol.

[21] Young ND, Debelle F, Oldroyd GE, Geurts R, Cannon SB, Udvardi MK, Benedito VA, Mayer KF, Gouzy J, Schoof H (2011). The Medicago genome provides insight into the evolution of rhizobial symbioses. Nature.

[22] Schmutz J, Cannon SB, Schlueter J, Ma J, Mitros T, Nelson W, Hyten DL, Song Q, Thelen JJ, Cheng J (2010). Genome sequence of the palaeopolyploid soybean. Nature.

[23] Ronquist F, Teslenko M, van der Mark P, Ayres DL, Darling A, Hohna S, Larget B, Liu L, Suchard MA, Huelsenbeck JP (2012). MrBayes 3.2: efficient Bayesian phylogenetic inference and model choice across a large model space. Syst Biol.

[24] Varshney RK, Chen W, Li Y, Bharti AK, Saxena RK, Schlueter JA, Donoghue MT, Azam S, Fan G, Whaley AM (2011). Draft genome sequence of pigeonpea (Cajanus cajan), an orphan legume crop of resource-poor farmers. Nat Biotechnol.

[25] Varshney RK, Song C, Saxena RK, Azam S, Yu S, Sharpe AG, Cannon S, Baek J, Rosen BD, Tar’an B (2013). Draft genome sequence of chickpea (Cicer arietinum) provides a resource for trait improvement. Nat Biotechnol.

[26] Schmutz J, McClean PE, Mamidi S, Wu GA, Cannon SB, Grimwood J, Jenkins J, Shu S, Song Q, Chavarro C (2014). A reference genome for common bean and genome-wide analysis of dual domestications. Nat Genet.

[27] Istvanek J, Jaros M, Krenek A, Repkova J (2014). Genome assembly and annotation for red clover (Trifolium pratense; Fabaceae). Am J Bot.

[28] Young ND, Mudge J, Ellis TH (2003). Legume genomes: more than peas in a pod. Curr Opin Plant Biol.

[29] Gracy J, Le-Nguyen D, Gelly J-C, Kaas Q, Heitz A, Chiche L (2008). KNOTTIN: the knottin or inhibitor cystine knot scaffold in 2007. Nucleic Acids Res.

[30] Cronk Q, Ojeda I, Pennington RT (2006). Legume comparative genomics: progress in phylogenetics and phylogenomics. Curr Opin Plant Biol.

[31] Da Silva P, Rahioui I, Laugier C, Jouvensal L, Meudal H, Chouabe C, Delmas AF, Gressent F (2010). Molecular requirements for the insecticidal activity of the plant peptide Pea Albumin 1 subunit b (PA1b). J Biol Chem.

[32] Rahioui I, Laugier C, Balmand S, Da Silva P, Rahbé Y, Gressent F (2007). Toxicity, binding and internalization of the pea-A1b entomotoxin in Sf9 cells. Biochimie.

[33] El Yahyaoui F, Kuster H, Ben Amor B, Hohnjec N, Puhler A, Becker A, Gouzy J, Vernie T, Gough C, Niebel A (2004). Expression profiling in Medicago truncatula identifies more than 750 genes differentially expressed during nodulation, including many potential regulators of the symbiotic program. Plant Physiol.

[34] Gamas P, Niebel FC, Lescure N, Cullimore J (1996). Use of a subtractive hybridization approach to identify new *Medicago truncatula* genes induced during root nodule development. Mol Plant-Microbe Inter.

[35] Hu L, Liu S (2011). Genome-wide identification and phylogenetic analysis of the ERF gene family in cucumbers. Genet Mol Biol.

[36] Boutrot F, Chantret N, Gautier MF (2008). Genome-wide analysis of the rice and Arabidopsis non-specific lipid transfer protein (nsLtp) gene families and identification of wheat nsLtp genes by EST data mining. BMC Genomics.

[37] Olsen KM, Wendel JF (2013). A bountiful harvest: genomic insights into crop domestication phenotypes. Annu Rev Plant Biol.

[38] Hanada K, Hirano H (2004). Interaction of a 43-kDa receptor-like protein with a 4-kDa hormone-like peptide in soybean. Biochemistry.

[39] Hanada K, Nishiuchi Y, Hirano H (2003). Amino acid residues on the surface of soybean 4-kDa peptide involved in the interaction with its binding protein. Eur J Biochem.

[40] Yamazaki T, Takaoka M, Katoh E, Hanada K, Sakita M, Sakata K, Nishiuchi Y, Hirano H (2003). A possible physiological function and the tertiary structure of a 4-kDa peptide in legumes. Eur J Biochem.

[41] Watanabe Y, Barbashov SF, Komatsu S, Hemmings AM, Miyagi M, Tsunasawa S, Hirano H (1994). A peptide that stimulates phosphorylation of the plant insulin-binding protein. Isolation, primary structure and cDNA cloning. Eur J Biochem.

[42] Dun XP, Wang JH, Chen L, Lu J, Li FF, Zhao YY, Cederlund E, Bryzgalova G, Efendic S, Jornvall H (2007). Activity of the plant peptide aglycin in mammalian systems. FEBS J.

[43] Poth AG, Colgrave ML, Lyons RE, Daly NL, Craik DJ (2011). Discovery of an unusual biosynthetic origin for circular proteins in legumes. Proc Natl Acad Sci U S A.

[44] Nguyen GK, Zhang S, Nguyen NT, Nguyen PQ, Chiu MS, Hardjojo A, Tam JP (2011). Discovery and characterization of novel cyclotides originated from chimeric precursors consisting of albumin-1 chain a and cyclotide domains in the Fabaceae family. J Biol Chem.

[45] Cardoso D, de Queiroz LP, Pennington RT, de Lima HC, Fonty E, Wojciechowski MF, Lavin M (2012). Revisiting the phylogeny of papilionoid legumes: New insights from comprehensively sampled early-branching lineages. Am J Bot.

[46] Ilgoutz SC, Knittel N, Lin JM, Sterle S, Gayler KR (1997). Transcription of genes for conglutin gamma and a leginsulin-like protein in narrow-leafed lupin. Plant Mol Biol.

[47] Lavin M, Herendeen PS, Wojciechowski MF (2005). Evolutionary rates analysis of Leguminosae implicates a rapid diversification of lineages during the tertiary. Syst Biol.

[48] Marsolais F, Pajak A, Yin F, Taylor M, Gabriel M, Merino DM, Ma V, Kameka A, Vijayan P, Pham H (2010). Proteomic analysis of common bean seed with storage protein deficiency reveals up-regulation of sulfur-rich proteins and starch and raffinose metabolic enzymes, and down-regulation of the secretory pathway. J Proteome.

[49] Yin F, Pajak A, Chapman R, Sharpe A, Huang S, Marsolais F (2011). Analysis of common bean expressed sequence tags identifies sulfur metabolic pathways active in seed and sulfur-rich proteins highly expressed in the absence of phaseolin and major lectins. BMC Genomics.

[50] Legocki RP, Verma DP (1980). Identification of “nodule-specific” host proteins (nodoulins) involved in the development of rhizobium-legume symbiosis. Cell.

[51] Eyraud V, Karaki L, Rahioui I, Sivignon C, Da Silva P, Rahbe Y, Royer C, Gressent F (2013). Expression and biological activity of the cystine knot bioinsecticide PA1b (Pea Albumin 1 Subunit b). PLoS One.

[52] Heitz A, Chiche L, Le-Nguyen D, Castro B (1989). 1H 2D NMR and distance geometry study of the folding of Ecballium elaterium trypsin inhibitor, a member of the squash inhibitors family. Biochemistry.

[53] Craik DJ, Malik U (2013). Cyclotide biosynthesis. Curr Opin Chem Biol.

[54] Mitchell A, Chang HY, Daugherty L, Fraser M, Hunter S, Lopez R, McAnulla C, McMenamin C, Nuka G, Pesseat S (2015). The InterPro protein families database: the classification resource after 15 years. Nucleic Acids Res.

[55] Finn RD, Bateman A, Clements J, Coggill P, Eberhardt RY, Eddy SR, Heger A, Hetherington K, Holm L, Mistry J (2014). Pfam: the protein families database. Nucleic Acids Res.

[56] Guefrachi I, Nagymihaly M, Pislariu CI, Van de Velde W, Ratet P, Mars M, Udvardi MK, Kondorosi E, Mergaert P, Alunni B (2014). Extreme specificity of NCR gene expression in Medicago truncatula. BMC Genomics.

[57] Mergaert P, Nikovics K, Kelemen Z, Maunoury N, Vaubert D, Kondorosi A, Kondorosi E (2003). A novel family in *Medicago truncatula* consisting of more than 300 nodule-specific genes coding for small, secreted polypeptides with conserved cysteine motifs. Plant Physiol.

[58] Altschul SF, Madden TL, Schäffer AA, Zhang J, Zhang Z, Miller W, Lipman DJ (1997). Gapped BLAST and PSI-BLAST: a new generation of protein database search programs. Nucleic Acids Res.

[59] Bru C, Courcelle E, Carrere S, Beausse Y, Dalmar S, Kahn D (2005). The ProDom database of protein domain families: more emphasis on 3D. Nucleic Acids Res.

[60] Eddy SR (2011). Accelerated Profile HMM Searches. PLoS Comput Biol.

[61] Bendtsen JD, Nielsen H, von Heijne G, Brunak S (2004). Improved prediction of signal peptides: SignalP 3.0. J Mol Biol.

[62] Bairoch A, Apweiler R, Wu CH, Barker WC, Boeckmann B, Ferro S, Gasteiger E, Huang H, Lopez R, Magrane M (2005). The Universal Protein Resource (UniProt). Nucleic Acids Res.

[63] Katoh K, Standley DM (2013). MAFFT multiple sequence alignment software version 7: improvements in performance and usability. Mol Biol Evol.

[64] Guindon S, Dufayard JF, Lefort V, Anisimova M, Hordijk W, Gascuel O (2010). New algorithms and methods to estimate maximum-likelihood phylogenies: assessing the performance of PhyML 3.0. Syst Biol.

[65] Le SQ, Gascuel O (2010). Accounting for solvent accessibility and secondary structure in protein phylogenetics is clearly beneficial. Syst Biol.

[66] Yang Z (1997). PAML: a program package for phylogenetic analysis by maximum likelihood. Comput Appl Biosci.

[67] De Mita S, Siol M (2012). EggLib: processing, analysis and simulation tools for population genetics and genomics. BMC Genet.

[68] Romiguier J, Figuet E, Galtier N, Douzery EJ, Boussau B, Dutheil JY, Ranwez V (2012). Fast and robust characterization of time-heterogeneous sequence evolutionary processes using substitution mapping. PLoS One.

[69] Sievers F, Wilm A, Dineen D, Gibson TJ, Karplus K, Li W, Lopez R, McWilliam H, Remmert M, Soding J (2011). Fast, scalable generation of high-quality protein multiple sequence alignments using Clustal Omega. Mol Syst Biol.

[70] Da Silva P, Strzepa A, Jouvensal L, Rahioui I, Gressent F, Delmas AF (2009). A folded and functional synthetic PA1b, an interlocked entomotoxic miniprotein. Biopolymers.

