# Supplementary material for: Genome-wide analysis identifies gain and loss/change of function within the small multigenic insecticidal Albumin 1 family of Medicago truncatula
Source: BMC Plant Biol. 2016 Mar 10;16:63. doi: 10.1186/s12870-016-0745-0 (PMC4785745; doi:10.1186/s12870-016-0745-0)
Supplement: Additional file 2: Table S5. — Position-numbered alignment of all Medicago truncatula A1b proteins, grouped and colored by identified clusters/clades (see Fig. 3) (DOCX 87 kb) [file 12870_2016_745_MOESM2_ESM.docx]

**10        20        30        40        50        60        70        80        90        100       110       120**

**|         |         |         |         |         |         |         |         |         |         |         |**

Medtr6g017150__1@ **--------MA-YVRLIPL--VVFLLAIF-STFSMKKLVIA-ADCSGI---CSPFE-----MPPC-RSS-----DCR-CIP-IA----LIGGFCINPIS-------SIMKMVEEHPNL-CQ**Medtr8g022430__1@ **--------MA-YIRFAHL--VVFLLAAF-SLVPTKK-VGA-TDCSGA---CSPFE-----MPPC-RSS-----DCR-CIP-IG----LVAGYCTYPSSP------TVMKMVEEHPNL-CQ**

Cluster 1

Medtr6g017170__1@ **--------MT-YVRLAPL--VVFLFATL-LMFLMKK-VGA-DECWGP---CSVLQ-----TPPC-PLS-----KCY-CIP-LF----LVVGYCSHASSP------TVMKMVEEHPNL-CQ**Medtr8g022400__1@ **--------MA-YLRLAHL--VVFLLATFSLIFPMMK--AA-EDCLGI---CSPFE-----MPPC-PSS-----SCR-CIP-VI----LIGGNCVDPSSP------TITKMVEKHANL-CQ**Medtr8g022420__1@ **--------MA-YLRLAHL--VVFLLATF-SIFPMMK-GAE--ECSGI---CSPFQ-----MPPC-PSS-----NCR-CIP-LV----LAGGNCVDPSSP------TITKMVEKHANL-CQ**AJ574790_______2@ **--------MA-YLRLAHL--VVFLHATFSLIFPMMK--AA-EDCSGI---CSPFE-----MPPC-PSS-----SCR-CIP-VI----LIGGNYVDPSSP------TITKMVEKHANL-CQ**

Medtr4g026590__1@ **--------MA-YAKLPLL--VLFLLATFFAVFPMKK-VEA---CESH--LCWAWQ----PDKVC-YSGSEREPGCY-CHP-ET----FLNGFCRDYISEH-----DMMKKMEEDPNL-CQ**

Medtr3g463570__1@ **--------MA-HVKLAPFA-AVFLLAAF-LMFPMKK-VEA-ADCLIS---CSPDS------PHC-DV------GCE-CHI-TS----LFSGSCFSKFLV------DPKKMVEKYSKV-CQ**

Medtr3g067830__1@ **--------MS-NVKLASF--AVLLLAAF-LMFPMKK-VEA-KECRGD---CGTFL------GLC-AS------GCK-CVT-HDYVYGCYVGTCENPNLVS----GNFQRKVEE-PKL-CW**Medtr3g067430__1@ **--------MA-YVKLASF--AVFLLAAF-VMFPMKK-VEG-AGCQQS---CGTFL------PGC-R------NDCE-CVP-VD----CTYGVCVGFCENDNVVSGNFHRKVEEHPKL-CM**

Medtr3g067445__1@ **--------MA-YVKLAPF--AVFLLAAF-LMFPMNK-VEG-AGCQQS---CGTFL------PGC-R------NGCE-CVL-VD----CTFGVCVGFCEKDNVVSGNFHRKVDEHPKL-CM**Medtr3g067535__1@ **--------MT-YVKLAPF--AVFMLAAF-LIFPMKK-VEA-AFCFRV--PCNPD-------YGC-NG------DCV-CALTWH--PVVPMYECYDPRSYA-----ELKKKVEEPPKL-CW**Medtr3g067550__1@ **---------------------------------MKK-VEA-DKCGAY---CPYPR------LYC-SG------DCD-CEPFIASLPPRLNFKCVTPHSSA-----ELKKKVEEQPKL-CW**Medtr3g067540__1@ **--------MT-YVKLAPL--AVFLLAAF-LIFPMKK-VEA-DKCGAY---CPYPR------LYC-SG------DCD-CEPFIASLPPRLNFKCVTPHSSA-----ELKKKVEEQPKL-CW**

Cluster 6

Medtr3g067555__1@ **--------MT-YVKLAPL--AVFLLAAF-LIFPMKK-VEA-DKCGAY---CPYPR------LYC-SG------DCD-CEPFIASLPPRLNFKCVTPHSSA-----ELKKKVEEQPKL-CW**

Medtr3g067570__1@ **---------------------------------MKK-IEG-NSLSF----CSPTV-----ETGC-DY------GCY-CFI-CT---SWGLGNCEPLS--------TIKKKVEEQPNL-CW**Medtr3g067510__1@ **--------MA-YVKIAPF--AVFLLAAF-LMFSMKK-IEG-AKCGEA--CDTQF-------NFC-NAGD----GCR-CFI-TD-AYLTLPGFCAQLT--------SIEKKVEELPNL-CW**

Medtr3g067580__1@ **MVLSFDQSMA-NVKLAPF--AIFLLAAF-LMFSMKK-IEG-AKCGED---CDTQV------NFC-NNHD----GCQ-CFV-TG--VLNQPGWCATLL--------HIEKKVEEQPNL-CW**

Medtr3g067500__1@ **---------------------------------MKK-IEG-TGCGMP---CNEVH------DVC-DSE-----DCF-CYS-EA--SFSGDGNCVTFT--------PFIKKVEEHPNL-CQ**

Medtr3g067437__1@ **--------MA-NVKLAPF--AVFLLAAF-LMFPMKK-IEG-ESCESR--GCIFYI-----NDSC-PS------GCV-CDP-ID--PVTWAGVCVSYS--------SIKKKVEEHPNY-CE**

Medtr3g067270__1@ **--------MA-SVKLAPF--AVFLLAAF-LMFPMKK-VEG-VDCTGA--YCDDLT-------GC-GD------YCF-CDV-IY--FLGNQGVCVPYS--------AMKKKVEENPNL-CQ**

Medtr3g467750__1@ **--------MA-SVKLAPF--AVFLLAAF-LMFPMKK-VEG-VDCTGA--YCDDLT-------GC-GD------YCF-CDV-IY--FLGNQGVCVPYS--------AMKKKVEENPNL-CQ**

Medtr3g067280__1@ **--------MA-SVKLAPF--AVFLLAAF-LMFPMKK-VEG-VDCTGV--YCDQFT-------DC-AE------ECF-CEV-IY--FIGSEGICYPYN--------TMKKKVEENPNL-CQ**

Medtr3g467760__2@ **--------MA-SVKLAPF--AVFLLAAF-LMFPMKK-VEG-VDCTGE--Y*DKIT-------DC-EE------KCF-CEV-IY--FIGNKGICNPYN--------TMKKKVEENPNL-CQ**

Medtr6g036620__1@ **--------MA-YFKLASL--AVFLLATF-LMFPTKN-VEA-QSCSGA--VCIRFN------TEC-DA------GCY-CHT-AG---TEQTGVCRPNV--------DGMEMEERHPYL-CQ**

Cluster 5

Medtr7g056803__1@ **--------MA-NAKLAPL--AVFLLVTF-LMISMKK-VEA-QSCGGA---CAVFD----SNPKC-GSS-----KCK-CVY-SI--IPFIAGHCDVRSST------D-VETDEEHPNL-CR**

Medtr6g082060__1@ **--------MA-YAKLAPL--VVFLFATF-LMFPMEN-VEA-QSCLSA---CTIFG----SKPLC-VSS-----RCRHCCK-MP--LNSFVGLCERRLST------DSERXEEEYPNYFCE**

Medtr7g056817__1@ **--------MS-YAKLTPL--ALFLFATF-LMFPMKK-VEA-QSCIGF---CSVFD----SKPLC-GSS-----RCR-CNK-PL--NNPFVGICERRPST------DAIEMEEEHQKF-CQ**

Medtr1g019650__1@ **--------MA-YAKLIPL--AVFMLATF-LMLPMKK-VEA--SCKDN--ICVGFF------LRC-GD------RCF-CYPQIG---------CVEQI------------IGENHPNI-CH**Medtr6g047880__1@ **--------MT-YLKLTPL--AVFFLATL-IILPMKK-VEA-SRCLMA--LCSATS------QTC-GE------GCF-CFP-TD--PWQGIYDCQPASYK------DLVKISGKNPNF-CQ**Medtr6g047900__1@ **--------MT-YAKLATL--AVFFLATL-IIFPMKK-VEA-DRCAMA--VCSDSL------KTC-GSV-----YCV-CIP-NE-------YRCLPASYK------DLVKIPGKNPNY-CQ**Medtr7g029540__1@ **--------MT-YVKHAPLALAVFLLATL-IMFPVKK-VEA-HHCSIG--PCSTPW------ATC-GSE-----YCI-CIP-MG-----SSNICQPSSYK------DVVKITGKNHNF-CQ**

Cluster 4

Medtr7g044920__1@ **--------MA-SVKLA-L--AVIFLAAF-IIFPMKK-VEA---CSGG--LCSVFD---KNFPRC-EENP----DCQ-CIP-WG----ILYGNCIYLPTKE-----SIVKVVEEHPNL-CQ**Medtr8g461020__1@ **--------MKPYLSCVNV--AVFVFRFS-VMFPMKK-AEALPECDKK--ECNRWK------EHC-SK------ECL-CIE-LF----MGIGYCYPIP--------TAMKKLKEHPNL-CQ**Medtr7g044980__1@ **--------MA-YLKFA-L--AAVFLATI-FICPMKK-VEA---CAAT--WCTVVE-----TTVC-GS------GCG-CLA-WG----IFGGNCVPRS--------SLTKMVEEHPNL-CQ**Medtr8g056800__1@ **---------------------------------MRN-VTA------------------------------------------G----GLAGFCRPQL--------TITKAVEEHYNL-SQ**

Cluster 2

TA24778_3880____@ **--------MT-YVKLAIL--AVLHLTIF-LIFQTKN-VEA-ASCPNVGAVCSPFE-----TKPC-GNVK----DCR-CLP-WG----LFFGTCINPTGSK-----YNMKMIEEHPNL-CQ**Medtr3g436100__1@ **--------MT-YVKLITL--ALFLVTTL-LMFQTKN-VEA-EFCSSVGSFCSPFN----T-NPC-GYLG----NCR-CVP-YY----LYGGTCENPFGFE-----HNMKMIEEHPNL-CQ**Medtr3g436120__2@ **--------MT-YVNLITL--ALFL---------TKN-VEA-LTCPNSGSLCSPFE----V-PAC-GNTI----DCR-CVP-YF----LFGGICHSPTSS----------LNE*HPNL-CQ**Medtr5g464490__1@ **--------MT-YVKLVTL--AVFMLTTF-LIVQTKN-VEA-GKCPSAGMVCSPFN-----PNQC-GNVI----QCR-CIP-GF---VIEAGICGDNFT----------------------**Medtr0112S0040_1@ **--------MT-YVKLVTL--AVFMLTIF-LIIQTKN-VEAAGQCPSVGSMIDMSQKLKT------------------------------NHACYPP------------------------**Medtr5g464350__1@ **--------MT-YVKLATL--AVFMLTTF-LIVQTKN-VEA-GECPSVGRGCTQLL-----LNPC-GNIL----ECI-CVS-RW---IYGGSICQSL------------------------**Medtr5g464390__1@ **--------MT-YVKLATL--AVFMLTIF-LIVQTKN-VQA-GQCPSAGRSCYQLS----P-NAC-GDIE----ECI-CHS-EW---LYDGGICKTLY-----------------------**Medtr5g464590__1@ **--------MT-YVKLATL--AVFMLTQFCLIVQTKN-VEE-GQCPFAGRVCSQYE-----SNAC-GDSE----ECI-CVS-EW--SHYDGGICKSRN-----------------------**Medtr0093S0090_1@ **--------MT-YVKLVTL--AVFMLTTF-LIVETMN-IEA-RLCPTAGTACSQRR-----GNSC-GGI-----ECI-CVS-QG--YPYDGGICKSRN-----------------------**AC146565_34____1@ **--------MT-YVKLVTL--AVFMLTTF-LIVQIKN-VEA-GQCARVGMRCSRAL-----PNPCGGDII----TCR-CVR-LH----LIGSTCVDYTGDG-----L--------------**Medtr0416s0030_1@ **--------MT-YVKLANL--AVFMLTTF-LIVQIKN-VEA-GQCARVGMRCSRAL-----PNPC-GDIV----TCR-CVH-LH----LVGSTCIDYTGDG-----L--------------**AC146565_12____1@ **--------MT-YVKLATL--AVFMLTQFCLIVQIKN-VEA-GQCARVGMRCSRAL-----PNPC-GDIV----TCR-CVH-LH----LVGSTCIDYTGDG-----L--------------**AC146565_18____1@ **--------MT-YVMLVTL--AIFMLTTF-LIVQIKN-VEA-GQCARVGMRCSRAL-----PNPC-GDIV----TCR-CVH-LH----LVGSTCIDYTGDG-----L--------------**Medtr3g438140__1@ **--------MT-YVKLATL--AVFMLTTF-LIVQMKN-VEA-RHCARYGTKLRPLR-----------------------------------------------------------------**Medtr3g438170__1@ **--------MT-YVKLATL--AVFMLTTF-LIVQMKN-VEA-RHCARYGTKLRPLR-----------------------------------------------------------------**Medtr5g464540__1@ **--------MT-YVKLANL--AVFMLTTF-CI-QTKN-VEA-RHCARYGMPCAVFE-----SNPC-GNSI----TCQ-CVS-LY----IFGMSCIDYTGDG-----L--------------**

Cluster 3

Medtr0112s0050_1@ **--------MT-YVKLVTS--AVFMLTTF-LIIQTKN-VEA--YCARYGMRCSIFE-----LNPC-GNSI----TCQ-CVS-LQ----IFGMTCIGV-PDG-----L--------------**

**130       140       150       160       170       180       190**

**|         |         |         |         |         |         |**

Medtr6g017150__1@ **SHVDC-TKKKSGSFCARYPNPNIEYGWYFASNSEAR-DVF-----------FNISSNSE-LTKDLLKMHSTTYY------**Medtr8g022430__1@ **SHADC-TKKESGSFCARYPNPDIEHGWCFSSNFEAY-DVF-----------FNVSSNRG-LIKDFLPMFTLTLDS-----**Medtr6g017170__1@ **SHADC-TKKGSGSFCARYPNLDIEYGWCFASNSKAQ-EVF-----------FEIFSNYE-FI------------------**Medtr8g022400__1@ **SHADC-TKKGSGSFCARYPNPDIEYGWCFSSSSRAQ-EVF-----------FEISSNPQ-FIKDLKMRPDTCRSSFNLLM**Medtr8g022420__1@ **SHADC-TKKGSGSFCARYPNPDIKYGWCFSSNSKAQ-EVF-----------FKISSNPR-FMEDLKIKPGTCGSFFY---**AJ574790_______2@ **SHADC-TKKGSGSFCARYPNPDIEYGWCFSSNSKAQ-EVF-----------FKISSNPR-FMEDLKIKPGTCGSFFY---**

Medtr4g026590__1@ **SHADC-KKKGSGSFCARNPNTDIKYGWCFLSGSHAQ-TA------------FRNALNSE-FENLSLKMPSEVST------**

Medtr3g463570__1@ **SHTDC-TKKGSGSFCGRYPNSILEYGWCFASESEAE-DIF-----------FKIASKSK-FSKDFLKRPITV--------**Medtr3g067830__1@ **SHDEC-TKKGSGNYCARFLNYDT--------------------------------------------PSVPE--------**Medtr3g067430__1@ **SHDEC-TKKGSGNYCAHFPNSNLKYGYCFASVSEAQ-DA------------YKIASSLK-FKKDFLKMSLPA--------**Medtr3g067445__1@ **SHDEC-TKKGSGNYCAHFPNSNLKHGFCFASVSEA---------------------------------------------**Medtr3g067535__1@ **SHAEC-TKKGSGNYCARFPN--LQYGLCFPSVSEAV-NA------------FKMASSLK-FEKDFLKMSLPA--------**Medtr3g067550__1@ **SHTEC-TEKGSGNYCARFPNSNLKYGLCFPSISEAV-NT------------FKMASSLK-FEKDFLKMSLPA--------**Medtr3g067540__1@ **SHTEC-TEKGSGNYCARFPNSNLKYGLCFPSISEAV-NT------------FKMASSLK-FEKDFLKMSLPA--------**Medtr3g067555__1@ **SHTEC-TEKGSGNYCARFPNSNLKYGLCFPSISEAV-NT------------FKMASSLK-FEKDFLKMSLPA--------**Medtr3g067570__1@ **SHDEC-TKKGSGNYCARFPNSDIKYGWCFASISEEE-DA-----------------------------------------**Medtr3g067510__1@ **SHAEC-IRKGSGNYCAHLPNSDIKYGFCFASISEAE-DV------------L--------LKRDFLKMSVSA--------**Medtr3g067580__1@ **SHAEC-IRKGSGNYCAHLPNSNIKYGFCFASISVAE-AT------------FKMAASTI-FKSNFLKMSVPA--------**Medtr3g067500__1@ **THTEC-TKKGSGNFCARFPNSNRKYGFCVAANSEAK-EA------------FKMASSSK-LKNYFLKMYVPA--------**Medtr3g067437__1@ **THTEC-TKKGSGNFCARFANSDIKYGWCFASISEAE-NA------------FKIASTSE-FKNDL---------------**Medtr3g067270__1@ **THTEC-KKKGSGNFCARHINSDVKYGFCFASFSEAQ-DA------------YKMAITSN-IKKDFLKIPGTTAY------**Medtr3g467750__1@ **THTEC-KKKGSGNFCARHINSDVKYGFCFASFSEAQ-DA------------YKMAITSN-IKKDFLKIPGTTAY------**Medtr3g067280__1@ **THTEC-KKKGSGNFCARHINSDVKYGFCFASFSEAQ-DA------------YKMAITSN-IKKDFLKIPGTTAY------**Medtr3g467760__2@ **THTEC-KKKGSGNFCARHINSDVKYGFCFASFSEAQ-DA------------YKMAITSN-IKKDFLKIPGTTAY------**

Medtr6g036620__1@ **SHDEC-NKKGSGSFCARSPNSDNKNGWCFASFSEAQ-EY------------FKFTAKYK-FKRDFLKMPITA--------**Medtr7g056803__1@ **SHVEC-TEKGIGSFCARYLDPDNQFGWCFASYSEAE-EY------------FKIASKYK-FTKEFLKLPITA--------**Medtr6g082060__1@ **SHMDCXQKKGIGSFCARYSNFDNEHGWCFASISEAE-HI------------FKIASQYL-VGRGGPKEPVGYLGMEIG--**Medtr7g056817__1@ **SHTDC-TERGIGTFCARYPNSDNEYGWCFASSSEAE-EY------------FKIASKYK-FTKEFLKMPITA--------**

Medtr1g019650__1@ **SHVEC-MNKGSGSFCAHSPILDVDYGWCFAYKSEAE-DL------------LKM-SGTA---------------------**Medtr6g047880__1@ **SHVEC-KEKGSGSFCARYPSPNVDYGLCVASLSEGE-DF------------FKIASKLT-VAKDFLTMLEIA--------**Medtr6g047900__1@ **SHVEC-KEKGRGSFCARYPSPNVDYGWCVASISEAE-DFF-----------FKLASKST-VTKDFLKMFEIA--------**Medtr7g029540__1@ **SHVEC-KEKGRGSFCARYPSSKVDYGRCVASISEEE-DF------------LRMSVIV----------------------**

Medtr7g044920__1@ **SHVEC-TKKGSGSFCARYPDSKNHIGWCFTSNAEAE-RYFEVGSNPAINNFFKNMISNS-NEKGFLKMPVEIST------**Medtr8g461020__1@ **SHMDC-INKGSGSFCSYYPNSEIQHGLCFTSKVEAERRY------------FEFEVLAN-AT--AKDMPMVVAAA-----**Medtr7g044980__1@ **SHIDC-IKKGNGSFCARYPNSDIEHGWCFTSNVEAE-RYFEILINPATNNFLKTVSHSI-NGNGFLKMPVEIAS------**Medtr8g056800__1@ **SNNDC-IKKGTGSLCAYYPNSELQHGWCFTSNVEAE-HYFEVLSNPAINNFLKNISYSN--GKGFLKMPVEIAT------**

TA24778_3880____@ **THGEC-IKKGSGNFCARYANADIEYGWCFVSVSEAE-RYFKIGSNTAVKSFFKIASKEKDYLKMALEIATEE--------**Medtr3g436100__1@ **THAEC-IKKGSGNFCARYANADVEYGWCFASVAEAE-RYFKIGSNTAVKSLFKIASKSK--EQDYLKMALEIAT------**Medtr3g436120__2@ **THAEC-IKKGSGNFCARYANDDIEYGWCFASVAEAE-RYFKIGSNTAVKNLFKIASKSK--EQDYLKMALEIAT------**Medtr5g464490__1@ **--------------------------------------------------------------------------------**Medtr0112S0040_1@ **--------------------------------------------------------------------------------**Medtr5g464350__1@ **--------------------------------------------------------------------------------**Medtr5g464390__1@ **--------------------------------------------------------------------------------**Medtr5g464590__1@ **--------------------------------------------------------------------------------**Medtr0093s0090_1@ **--------------------------------------------------------------------------------**AC146565_34____1@ **--------------------------------------------------------------------------------**Medtr0416S0030_1@ **--------------------------------------------------------------------------------**AC146565_12____1@ **--------------------------------------------------------------------------------**AC146565_18____1@ **--------------------------------------------------------------------------------**Medtr3g438140__1@ **--------------------------------------------------------------------------------**Medtr3g438170__1@ **--------------------------------------------------------------------------------**Medtr5g464540__1@ **--------------------------------------------------------------------------------**Medtr0112s0050_1@ **--------------------------------------------------------------------------------**
